# Analysis of mRNA-miRNA-lncRNA differential expression in prediabetes/type 2 diabetes mellitus patients as potential players in insulin resistance

**DOI:** 10.3389/fendo.2023.1131171

**Published:** 2023-05-08

**Authors:** Hebatalla Said Ali, Marwa Mostafa Kamel, Sara H. A. Agwa, Mohamed S. Abdel Hakeem, Mahmoud Shawky El Meteini, Marwa Matboli

**Affiliations:** ^1^ Medical Biochemistry and Molecular Biology Department, Faculty of Medicine, Ain Shams University, Abbassia, Cairo, Egypt; ^2^ Clinical Pathology, Medical Ain Shams Research Institute, Ain Shams University, Cairo, Egypt; ^3^ Institute of Immunology, University of Pennsylvania, Philadelphia, PA, United States; ^4^ Department of General Surgery, The School of Medicine, University of Ain Shams, Cairo, Egypt

**Keywords:** insulin resistance, pre-diabetes, type 2 diabetes mellitus, bioinformatics, biomarkers, non-coding RNA

## Abstract

**Introduction:**

Type 2 diabetes mellitus (T2DM) is a major global health concern. It usually develops gradually and is frequently preceded by undetectable pre-diabetes mellitus (pre-DM) stage. The purpose of this study was to identify a novel set of seven candidate genes associated with the pathogenesis of insulin resistance (IR) and pre-DM, followed by their experimental validation in patients’ serum samples.

**Methods:**

We used the bioinformatics tools and through a two-step process, we first identified and verified two mRNA candidate genes linked to insulin resistance molecular pathogenesis. Second, we identified a non-coding RNAs related to the selected mRNAs and implicated in the insulin resistance molecular pathways followed by pilot study for the RNA panel differential expression in 66 patients with T2DM, 49 individuals with prediabetes and 45 matched controls using real time PCR.

**Results:**

The levels of expression of TMEM173 and CHUK mRNAs, hsa-miR (-611, -5192, and -1976) miRNAs gradually increased from the healthy control group to the prediabetic group, reaching their maximum levels in the T2DM group (p <10-3), whereas the levels of expression of RP4-605O3.4 and AC074117.2 lncRNAs declined gradually from the healthy control group to the prediabetic group, reaching their lowest levels in the T2DM group (p <10-3). TMEM173, CHUK mRNAs, hsa_miR (-611 & -1976) and RP4-605O3.4 lncRNA were useful in distinguishing insulin resistant from insulin sensitive groups. miR_611 together with RP4-605O3.4 exhibited significant difference in good versus poor glycemic control groups.

**Discussion:**

The presented study provides an insight about this RNA based STING/NOD/IR associated panel that could be used for PreDM-T2DM diagnosis and also as a therapeutic target based on the differences of its expression level in the pre-DM and T2DM stages.

## 1 Introduction

Insulin resistance has been identified as a leading cause of major health problems such as diabetes, hyperlipidemia, hypertension, and cardiovascular disease (1, 2). Insulin resistance can begin years before type 2 diabetes mellitus is diagnosed, even before prediabetes is recognized (3). Diabetes mellitus (DM) is a common metabolic condition characterized by hyperglycemia, insulin resistance, and significantly decreased insulin production. In 2021, the International Diabetes Federation estimated that 537 million adults globally have diabetes, and by 2045, this number will increase to 783 million. Diabetes and its consequences were expected to cost 966 billion US dollars in 2021, a 316% rise over the previous 15 years (4). Notably, the MENA region is now identified as an emerging center of the T2DM epidemic. Egypt is one of the 21 IDF MENA region countries and territories with 4.5 million diabetics (5, 6). The major problem is that in the early stages of the disease, most patients do not have specific symptoms, and many cases may present suddenly with serious complications (7).

Prediabetes mellitus (pre-DM) is a metabolic condition preceding T2DM, characterized by higher than normal blood glucose levels but below the diabetes threshold. Globally, the number of individuals with prediabetes is rising; approximately 541 million adults have prediabetes and are at greater risk of developing type 2 diabetes (4). The pre-diabetes mellitus stage is mainly characterized by β-cell dysfunction and insulin resistance, which can exist prior to observable changes in blood glucose levels. It is suggested that the early stages of neuropathy, nephropathy, retinopathy, and macrovascular disease all begin in the pre-DM stage (8, 9).

In recent years, several molecular pathways that are involved in the development of prediabetes and T2DM have gained attention. One such pathway is the alteration of gut microbiota, which is known to play an important role in insulin resistance. Studies have shown that gut microbiota can raise plasma LPS levels and microbial DNA, which in turn aggravate tissue inflammation through the cGAS-STING and NOD-like receptor pathways (10, 11).

The cGAS–STING pathway has recently gained attention as a mediator of inflammation caused by various factors, including infection, cellular stress, and tissue damage because of its ability to detect cellular responses to both microbial and host-derived DNAs (12–14). STING is a transmembrane protein triggered by cytosolic self-derived nucleic acids and is encoded by *TMEM173*. Most of *TMEM173* variants result in JAK/STAT pathway-mediated INF production, which is involved in a variety of inflammatory disorders (15). Additionally, *TMEM173* is involved in oxidative stress and autophagy (16), both of which are strongly related to insulin signaling. This suggests that TMEM173 may be involved in the development of diabetes (17). *TMEM173* variants were also found in many human diseases such as esophageal squamous cell carcinoma (18), colon cancer (19), lung diseases including familial interstitial lung disease (20), and severe pulmonary fibrosis (21) and drives lethal coagulation in sepsis (22), These findings make TMEM173 a potential therapeutic target for both cancers and inflammatory diseases.

NOD-like receptors (NLRs) have been implicated in a number of human diseases. They play an important role in regulating inflammatory responses by inducing the production of chemokines, cytokines, and antimicrobial genes, which contributes to the development of obesity and T2DM (23). Pathogenic variants in the NLRs have been associated with alteration in the NF-κB signaling pathway, leading to an inability to respond properly. The *CHUK* gene is a member of the inhibitor of the nuclear factor kappa B kinase complex, encoding for inhibitor-κB kinase α that degrades IκBα and NF-κB inhibitors through phosphorylation, causing activation of NF-κB. CHUK/IKK has also been recognized as a tumor suppressor in multiple human and mouse organs (24). Furthermore, it has been linked to plasma lipid abnormalities, a major risk factor for stroke (25, 26).

Epigenetic dysregulation that could alter the expression of many target genes is another crucial player in insulin resistance and T2DM pathogenesis. Non-coding RNAs (ncRNAs) play an important role in cellular function and disease development. Their up- or downregulation affects glucose homeostasis and is associated with the development of diabetes. They are also associated with poor glycemic control, proinflammation and senescence in T2DM individuals (27). They are found to be good diagnostic biomarkers in several metabolic illnesses such as diabetes, obesity, and non-alcoholic fatty liver disease (NFLD) due to their stability in bodily fluids (28).

Several studies have found involvement and changes in expression of different miRNAs in processes implicated in the pathogenesis of type 2 diabetes (29, 30). *MiR-611* is located on chromosome 11q12.2, a region that is commonly altered or amplified in cancer. *miR-611* was found to be significantly dysregulated in tongue squamous cell carcinoma and was strongly linked with TNM stage (31). Regarding *miR-5192*, it was found to be differentially expressed in idiopathic pulmonary fibrosis (32), human breast cancer cells (33), and childhood acute lymphoblastic leukemia (34). *miR-1976* is located on 1p36.1; its aberrant expression has been related to the progression of breast cancer (35), and it may also predispose to Parkinson’s disease (36) and function as a predictive biomarker for obesity and weight loss (37).

lncRNAs have gained attention due to a greater understanding of their functional role in both health and disease. The extensive dysregulation of lncRNA expression in a variety of diseases emphasizes their importance as master regulators. Recent study reveals that lncRNAs play a role in disease development; they may affect glucose homeostasis and be associated with development of diabetes, and hence should be evaluated and targeted for therapeutic benefits (38).

Despite the advances in diagnosis and improvement in therapies, T2DM still remains an incurable disease. Increasing attention is focused on understanding the molecular mechanisms (e.g. STING, NOD signaling) that cause IR and complications that starts early in the prediabetes stage. This study aimed to identify novel pre-DM and T2DM related RNA panel that may aid in discrimination of risky pre-diabetes, DM-T2DM, and thus can delay or prevent the development of T2DM complications.

To achieve this objective, we identified a STING/NOD/IR RNA panel consisting of lncRNAs, miRNAs. We integrated differentially expressed mRNA-miRNA interactions with lncRNA-miRNA interactions using *in silico* data analysis followed by pilot study for the chosen circulatory RNA panel differential expression analysis in pre-diabetes, T2DM, and healthy control by real-time PCR.

## 2 Results

### 2.1 Bioinformatics results

The identification of the target genes and pathways followed a specific selection criterion, based mainly on bioinformatics-based selection and other criteria such as Pathway analysis, Relevance to the disease of interest, Functionality, Cost-effectiveness, and Accessibility. These criteria help to ensure that the target genes and pathways selected for the RNA PCR gene expression panel analysis are relevant, well-established, and feasible to measure, which can increase the confidence in the results and the impact of the study.

**Figure 1:**
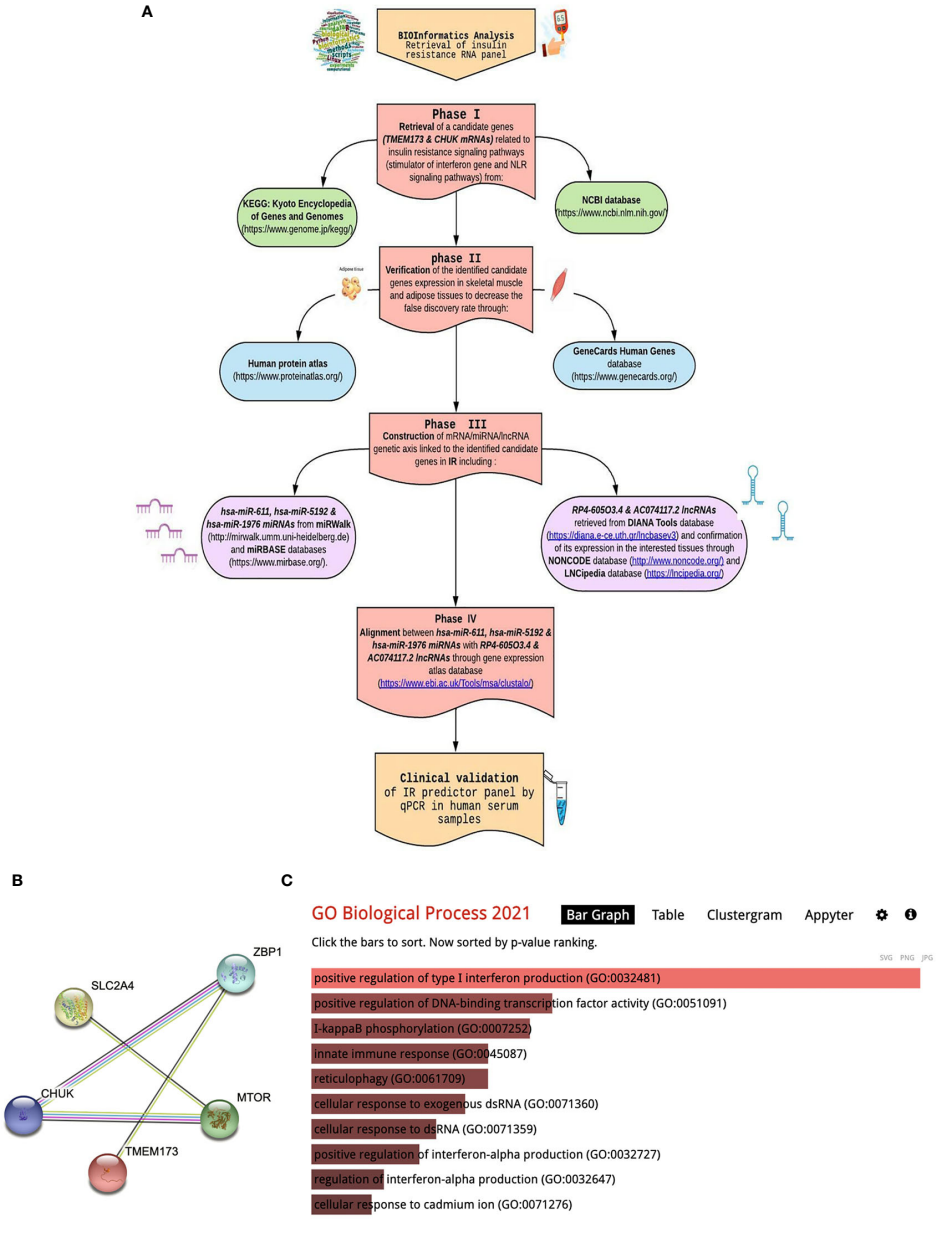
Bioinformatics workflow include: **(A)** Bioinformatics chart for retrieval of STING/NOD/IR RNA panel, **(B)** STRING interaction (STRING pathway analysis of significantly dysregulated biomarkers in T2DM). **(C)** Gene ontology enrichment analysis using the Enrichr algorithm (available at: https://maayanlab.cloud/Enrichr/).

We used bioinformatics analysis to select candidate mRNA genes which are related to Type 2 DM, insulin resistance signaling, STimulator of Interferon Gene (STING), and NLR signaling pathways from public microarray databases, e.g., National Center of Biotechnology Information GEO (https://www.ncbi.nlm.nih.gov/geo/, available May 2022), Gene atlas expression database (https://www.ebi.ac.uk/gxa/home), and KEGG map (https://www.genome.jp/kegg/) to choose a pathway related to insulin resistance in T2DM and identify the target effector. Search was confined to Homo sapiens species and p-value **≤**0.05 was considered statistically significant. Among 156 differentially expressed genes and FDR-adjusted p-value **<**0.05, We have retrieved a set *TMEM173* and *CHUK* mRNAs according to the following criteria: (i) Genes which are dysregulated in T2DM and insulin resistance molecular pathways; (ii) Genes which are expressed in tissues of interest, e.g., skeletal muscle, adipose tissue and also in blood samples for easy extraction and less invasiveness; (iii) Both genes are related to STING and NLR signaling pathway involved in innate immunity and chronic sterile inflammation; (iv). Gene ontology (GO) enrichment of *TMEM173* and *CHUK* mRNAs were performed using Enrichr (http://amp.pharm.mssm.edu/Enrichr) with p < 0.05 set as the cutoff criterion that verified their correlation to positive regulation of interferon production and kappa beta phosphorylation. *TMEM17*3 and *CHUK* mRNAs were imported into the Search Tool for the retrieval of interacting genes (STRING; version 11.0; http://stringdb.org) online database for protein–protein interaction (PPI) network building to ensure their link to insulin resistance pathways and previously known genes in T2DM through STRING interaction network. PPI enrichment p-value was 1.6E-05 with combined score 0.943 for *CHUK* and *TMEM173*, 0.847. Afterwards, we predicted upstream key miRNAs and lncRNAs using miRWalk 3.0 (http://mirwalk.umm.uni-heidelberg.de/) and DIANA Tools database, which integrate the target prediction of both TargetScan * MiRBase. miRNAs and lncRNAs interaction was predicted by using miRWalk 2.0; miRNA: ncRNA target tool. We found that *hsa-miR (-611*, *-5192*, and *-1976)* can interact with both selected mRNAs with score >0.9 at CDS binding sites. Finally, *RP4-605O3.4* and *AC074117.2* were identified to be interacting with the chosen *miR (-611*, *-5192*, and -*1976*) and which was confirmed by Clustal Omega tool of The European Bioinformatics Institute based on high complementarity rate between them. Thus, finally, the (*TMEM173* and *CHUK*) - (*miR-611*, *-5192* and-*1976*) - (*RP4-605O3.4* and *AC074117.2*) regulatory panel was constructed. Detailed bioinformatics are shown in **Figure 1** and **Supplementary Figures S1–9**.

### 2.2 Clinical and biochemical indices

The present study includes 160 study subjects, classified into 66 cases with type 2 diabetes mellitus, 49 were prediabetics and 45 healthy volunteers. There were no significant differences as regards sex, age, and smoking between the three investigated groups (*p* > 0.05). On the other hand, there were significant differences among the three studied groups concerning family history of T2DM (p < 0.001), fasting and 2-h post-prandial blood glucose levels (p < 0.001), HbA1c, fasting insulin level (p < 0.001), BMI (p < 0.001), systolic and diastolic BP (p < 0.001), lipid profile including total Cholesterol, LDLc, HDLc, and Triglycerides (p < 0.001), and Alb/Cr. ratio (p < 0.001) as shown in **Supplementary Table S1**.

HOMA-IR was used as a valid surrogate indicator of insulin resistance and was calculated according to the equation: Fasting insulin (μU/L) x fasting glucose (nmol/L)/22.5 (39), while HOMA-B was calculated as follows: (20x insulin in mIU/ml)*/*(glucose in mmol/L – 3.5) and it has been suggested to be an effective indicator of B-cell function (40). Both formulas measure HOMA-IR, although they do so from a different perspective. We found significant differences between the three studied groups concerning HOMA-IR (p = <0.001), which was significantly higher in T2DM group compared to prediabetic and healthy control groups; it was also higher in the prediabetic group compared to the healthy control group which indicates different degrees of insulin sensitivity between the three groups.

### 2.3 Spearman correlation analysis

There was high positive significant correlation between both mRNAs *TMEM173* and *CHUK* and the three miRNAs: *hsa-miR (-611, -5192*, and *-1976)* while it was a negative correlation with the two lncRNAs: *RP4-605O3.4* and *AC074117.2* among all the studied groups and also among the prediabetic and the healthy control group which suggests that the lncRNAs work as a ceRNA competing with the miRNAs for the mRNA pool in the molecular pathogenesis of IR, prediabetes, and T2DM (**Table S2-S4**).

**Figure 2:**
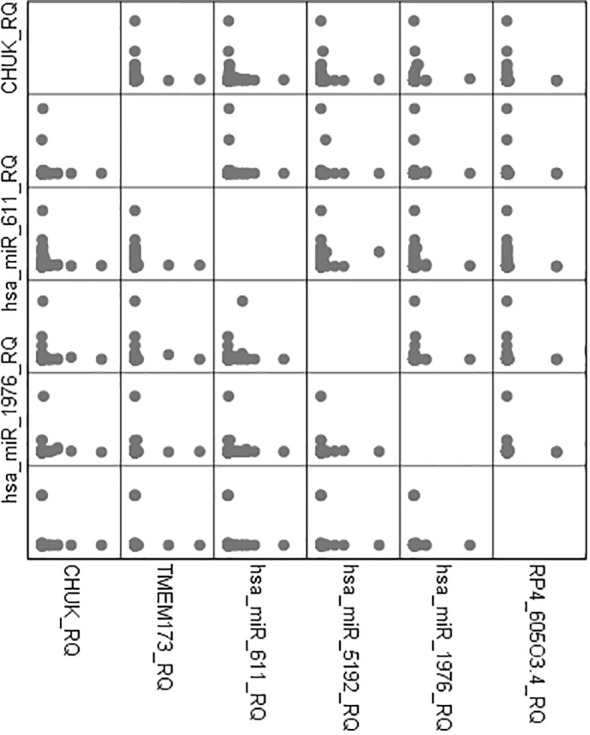
Graphical pairwise correlations between candidate genes.

**Figure 3:**
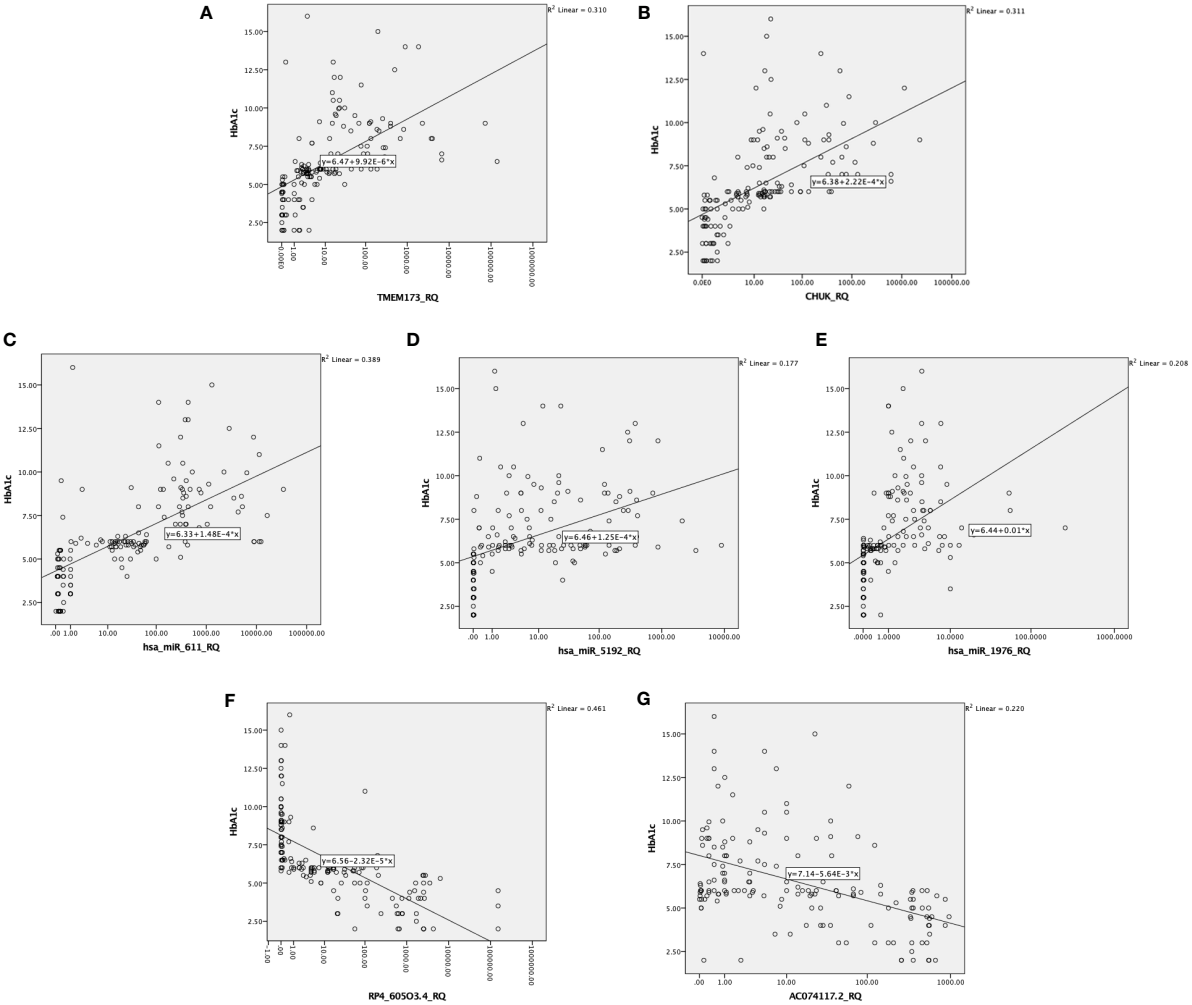
Correlation between the investigated IR predictor panel and glycemic control represented by HbA1c among all the studied groups (n = 160); as shown HbA1c is correlated with **(A, B)** the relative quantity of *TMEM-173* and *CHUK*, respectively, **(C–E)** the relative quantity of *hsa-miR (-611, -5192*, and *-1976)*, respectively, **(F, G)** the relative quantity of *RP4-605O3.4* and *AC074117.2*, respectively.

**Figure 4:**
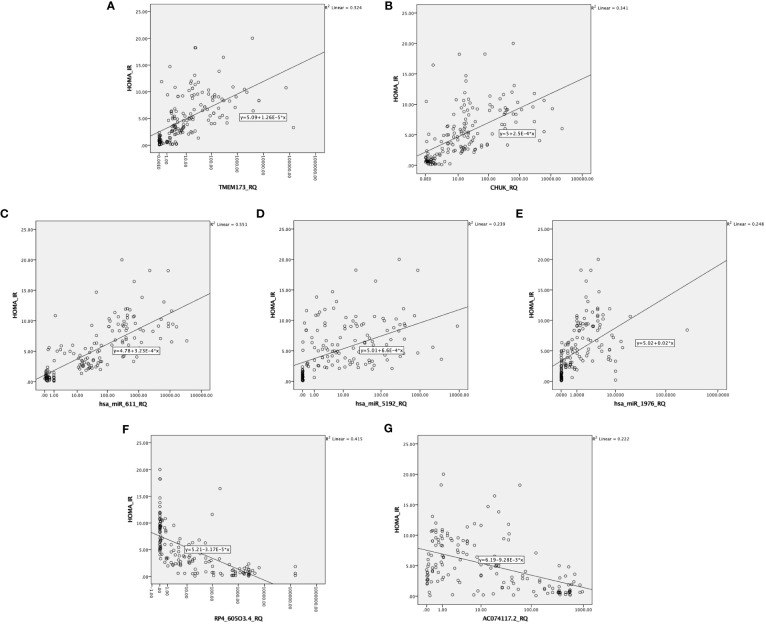
Correlation between the investigated IR predictor panel and degree of insulin resistance represented by HOMA-IR among all the studied groups (n = 160). As shown HOMA-IR is correlated with **(A, B)** the relative quantity of *TMEM-173* and *CHUK*, respectively. **(C–E)** the relative quantity of *hsa-miR (-611, -5192*, and *-1976)*, respectively, **(F, G)** the relative quantity of *RP4-605O3.4* and *AC074117.2*, respectively.

There was also a positive significant correlation between both mRNAs *TMEM173* and *CHUK*, the three miRNAs; *hsa-miR (-611, -5192*, and *-1976)* and the different clinicopathological factors except HOMA-B, which was negatively correlated, while the correlation was negative with the two lncRNAs; *RP4-605O3.4* and *AC074117.2* and the different clinicopathological factors but it was positive correlation with HOMA-B (**Table S4**). Spearman correlation was also done between the STING/NOD/IR related RNA associated panel among prediabetic and T2DM groups, there were significant positive correlations between the expression of *TMEM173* and *CHUK* mRNAs, *hsa-miR (-611* and *-1976)*, and negative correlation with *RP4-605O3.4* lncRNA. To evaluate the diagnostic potential of the investigated IR panel, Spearman correlation of the clinical traits such as: HbA1c and HOMA-IR, with the levels of expression of individual RNA was done for better modeling of how these RNA levels could explain the pathogenesis of IR/T2DM and to reveal the usefulness of these RNAs as diagnostic markers (**Figures 2**–**4** and **Tables S2–S4**).

### 2.4 Differential expression of the insulin resistance associated panel in the investigated groups

**Figure 5:**
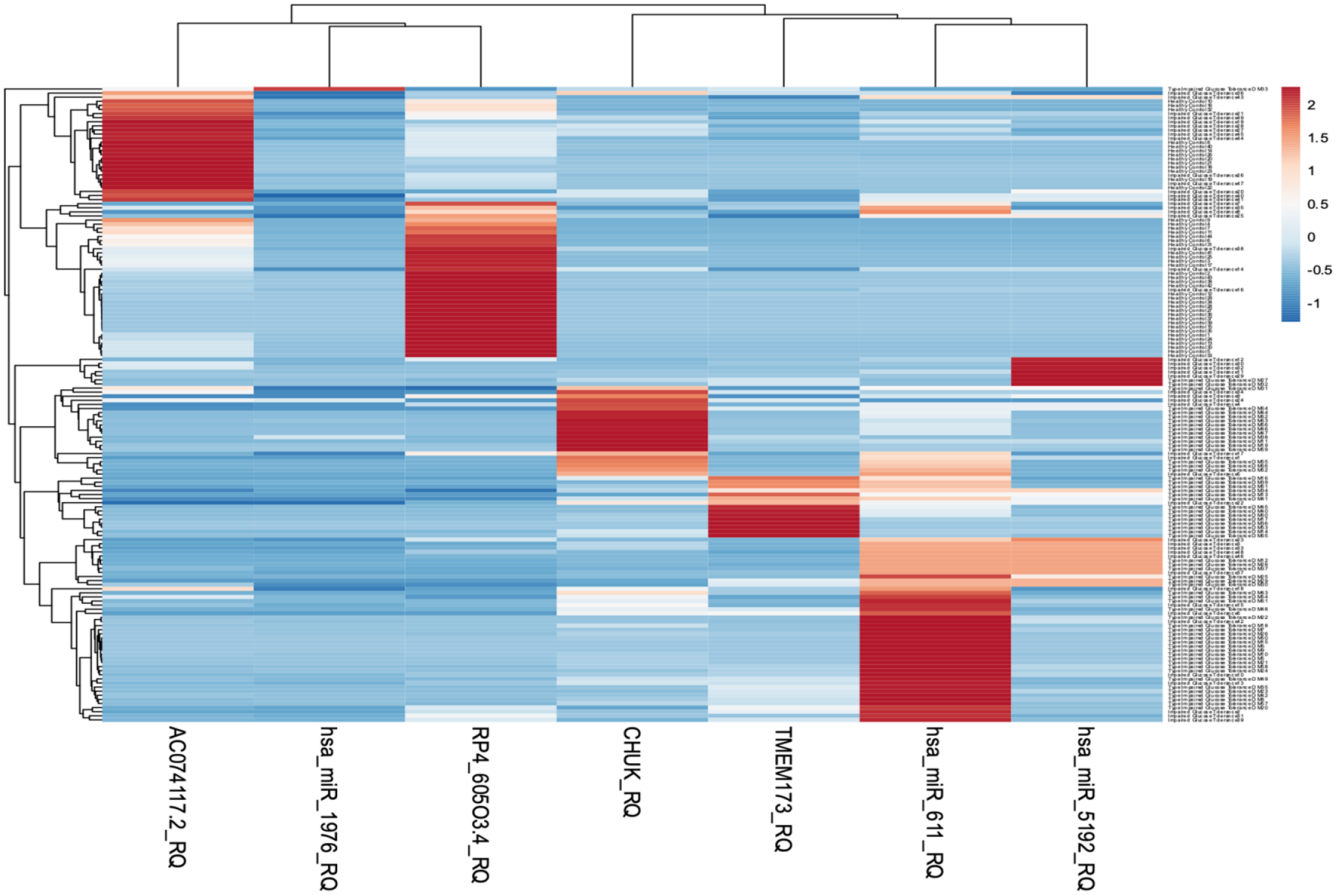
Heatmap representing hierarchal clustering of serum RNA expression was generated using the web tool ClustVis.

The heatmap (https://biit.cs.ut.ee/clustvis/) is generated using the web tool ClustVis representing hierarchal clustering of the associations between serum RNA expression and the selected clinical traits (41). It was a standardization with a mean at 0 and a standard deviation at 1. The differences were set as |FC| > 1.2 and p-value <0.05. Gene expression levels were illustrated as Z-scores (blue to red). Individuals are in rows, while transcripts are in columns. Blue color indicates lower expression while red color indicates high expression. Light brown color indicates downregulation while dark red color indicates upregulation (**Figure 5**).

**Figure 6:**
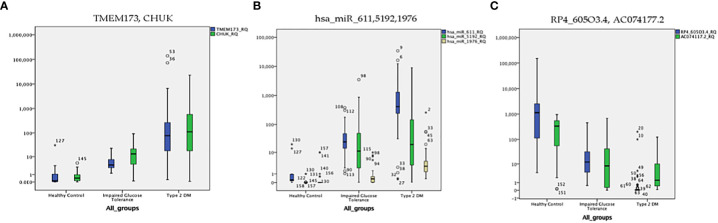
The distribution of the differential expression of the seven candidate genes (measured by qRT-PCR) in the three investigated groups; **(A)** *TMEM173 and CHUK* mRNAs, **(B)** *hsa-miR (-611, -5192*, and *-1976)* miRNAs, **(C)** *RP4-605O3.4* and *AC074117.2* lncRNAs. The median represented by the line inside the box, 1st and 3rd quartiles represented by the top and bottom lines of the box, respectively. Outliers represented by the dots; logarithmic scale Y axis = 10.

The level of expression of the investigated RNA panel was assessed in terms of fold-change (RQ) values in the three investigated groups (T2DM group, prediabetes group, and healthy control group) aiming to confirm the retrieved bioinformatics data. *TMEM173* and *CHUK* mRNAs, *hsa-miR (-611, -5192*, and *-1976)* miRNAs expression levels increased gradually from the control group to the pre-diabetic group to reach the highest levels in the T2DM group (p < 0.001), while *RP4-605O3.4* and *AC074117.2* lncRNAs expression levels decreased gradually from the control group to the pre-diabetic group to reach the lowest levels in the T2DM group (p < 0.001). Thus, the pattern of expression of the investigated STING/NOD/IR RNA associated panel was discriminative between prediabetes and T2DM groups compared to healthy controls and between prediabetes and T2DM patients (**Table 1** and **Figure 6**).

**Table 1:** The differential expression of the investigated STING/NOD/IR RNA associated panel among the 3 investigated groups with comparison in between the groups.

Group		Median	IQR	χ2	p
***TMEM173 RQ***	T2DM	77.50	18–266	KWχ2 = 114.187 ^a^ U = 144.00 ^b^ U = 271.00 ^c^	< 0.001* < 0.001* < 0.001*
	Impaired Glucose Tolerance	4.00	2.87–7.39		
	Healthy control	0.137	0.04–1.1		
***CHUK RQ***	T2DM	110.96	18–575.15	KWχ2 = 104.131^a^ U = 91.00^b^ U = 595.50^c^	< 0.001* < 0.001* < 0.001*
	Impaired Glucose Tolerance	13.13	4.4–21.5		
	Healthy control	0.450	0.14–0.98		
***hsa_miR_611 RQ***	T2DM	411.17	242.08–1293.59	KWχ2 = 120.633 ^a^ U = 33.00 ^b^ U = 279.50^c^	< 0.001* < 0.001* < 0.001*
	Impaired Glucose Tolerance	24.43	14.3–47.62		
	Healthy control	0.24	0.14–0.95		
***hsa_miR_5192 RQ***	T2DM	19.75	3.03–144.43	KWχ2 = 94.609 ^a^ U = 18.00 ^b^ U = 1403.00^c^	< 0.001* < 0.001* 0.226
	Impaired Glucose Tolerance	11.21	2.24–49		
	Healthy control	0.003	0.002–0.009		
***hsa_miR_1976 RQ***	T2DM	2.68	1.4–4.44	KWχ2 = 97.810 ^a^ U = 435.00 ^b^ U = 286.00 ^c^	< 0.001* < 0.001* < 0.001*
	Impaired Glucose Tolerance	.34	0.03–0.60		
	Healthy control	0.008	0.005–0.020		
***RP4-605O3.4 RQ***	T2DM	.0145	0.003–0.070	KWχ2 = 132.722 ^a^ U = 114.00 ^b^ U = 43.00 ^c^	< 0.001* < 0.001* < 0.001*
	Impaired Glucose Tolerance	12.00	4.23–32.02		
	Healthy control	1130.00	113–2536		
***AC074117.2 RQ***	T2DM	1.50	0.50–10.0	KWχ2 = 58.502 ^a^ U = 361.50 ^b^ U = 1360.00 ^c^	< 0.001* < 0.001* 0.146
	Impaired Glucose Tolerance	8.20	0.30–42.0		
	Healthy control	334.66	55.77–545.13		

^a^ Statistics in all groups; ^b^ Prediabetic versus healthy groups; ^c^ Prediabetic versus T2DM groups; KWχ2: Kruskal Wallis test, U: Mann–Whitney U test, After Bonferroni correction (*p < 0.007: Significant, p > 0.007: nonsignificant).

### 2.5 The performance characteristics of the insulin resistance associated panel in the investigated groups

**Figure 7:**
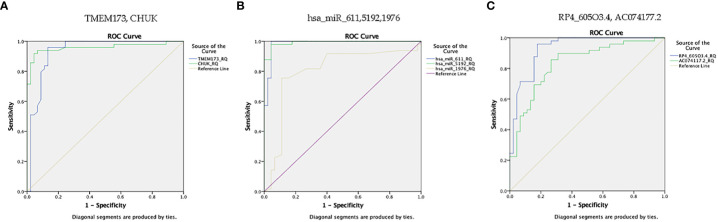
ROC curve represents the diagnostic accuracy of the investigated STING/NOD/IR RNA associated panel to discriminate between healthy controls and prediabetes group. **(A)** ROC curve analysis for serum *TMEM173* and *CHUK* mRNAs. **(B)** ROC curve analysis for serum *hsa-miR (-611, -5192*, and *-1976)* miRNAs. **(C)** ROC curve analysis for serum *RP4-605O3.4* and *AC074117.2* lncRNAs.

**Figure 8:**
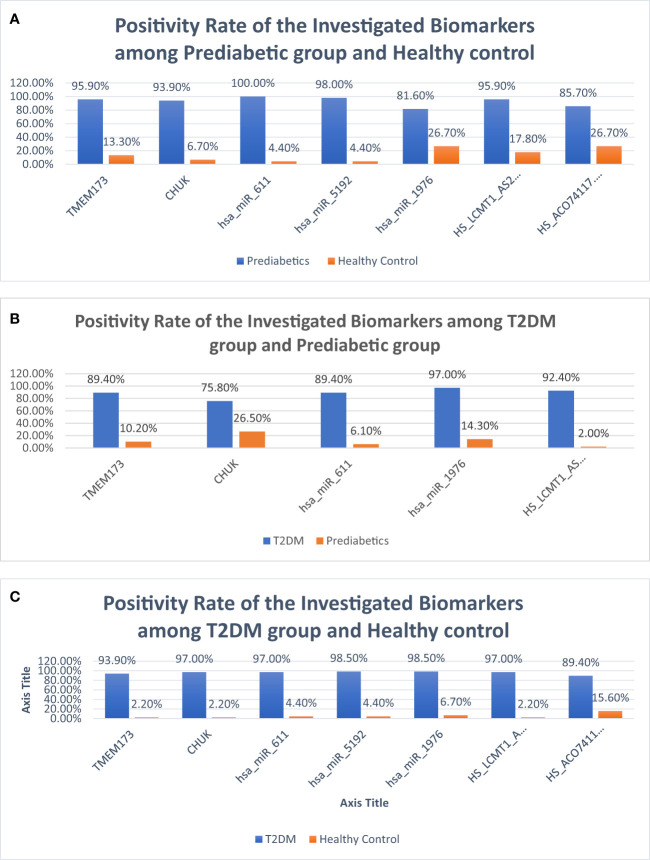
Bar charts demonstrating positivity rates of the investigated parameters: **(A)** between prediabetic and control groups, **(B)** between prediabetic and T2DM groups, and **(C)** between prediabetic and T2DM groups.

ROC curve analysis was performed for the investigated STING/NOD/IR RNA associated panel to discriminate between healthy controls and the prediabetes group and to determine the best cutoff values. As regards *TMEM173* and *CHUK* mRNAs, the ideal cutoff values were ≥1.80 (AUC=0.935) and ≥2.05 (AUC = 0.959), sensitivities were 95.9% and 93.9%, specificities were 86.7% and 93.3%, respectively. For serum *hsa-miR (-611, -5192*, and *-1976)* the best cutoff values were ≥1.018 (AUC = 0.985), ≥0.1654 (AUC = 0.992) and ≥0.0165 (AUC = 0.803), sensitivities were 100%, 98.0%, and 81.6%, specificities were 95.6%, 95.6%, and 73.3% respectively. Concerning serum *RP4-605O3.4* and *AC074117.2* lncRNAs the best cutoff values were ≤95 (AUC = 0.931) and ≤97.5 (AUC = 0.836), sensitivities were 95.9% and 85.7% specificities were 82.2% and 73.3%, respectively. Thus, the selected STING/NOD/IR RNA panel was associated with the prediabetes stage. Indeed, recognizing patients at risk for insulin resistance and prediabetes will assist clinicians to determine the optimal management strategy before developing T2DM (**Table 2** and **Figures 7**, **8**). Interestingly, the chosen RNAs could be used by clinicians to identify prediabetic patients especially those with family history of T2DM during routinely applied checking up.

**Table 2:** The performance characteristics of the insulin resistance associated panel among the investigated groups.

Performance Characteristics in Prediabetic Versus Healthy Control									
Test Result Variables	Area under Curve	Std. Error	p	95% Confidence Interval			Cutoff	Sensitivity	Specificity
				Lower Bound		upper Bound			
***TMEM173 RQ***	0.935	0.029	< 0.001*	0.879		0.991	1.800	95.9%	86.7%
***CHUK RQ***	0.959	0.022	< 0.001*	0.915		1.000	2.050	93.9%	93.3%
***hsa_miR_611 RQ***	0.985	0.011	< 0.001*	0.963		1.000	1.018	100.0%	95.6%
***hsa_miR_5192 RQ***	0.992	0.006	< 0.001*	0.980		1.000	0.1654	98.0%	95.6%
***hsa_miR_1976 RQ***	0.803	0.051	< 0.001*	0.704		0.902	0.0165	81.6%	73.3%
***RP4-605O3.4 RQ***	0.931	0.026	< 0.001*	0.880		0.982	95.00	95.9%	82.2%
***AC074117.2 RQ***	0.836	0.042	< 0.001*	0.754		0.918	97.50	85.7%	73.3%
Performance Characteristics in T2DM Patients Versus prediabetic									
***TMEM173 RQ***	0.916	0.029	< 0.001*	0.859		0.973	12.75	89.4%	89.8%
***CHUK RQ***	0.816	0.039	< 0.001*	0.740		0.892	17.74	75.8%	73.5%
***hsa_miR_611 RQ***	0.914	0.031	< 0.001*	0.853		0.974	105.39	89.4%	93.9%
***hsa_miR_1976 RQ***	0.912	0.034	< 0.001*	0.844		0.979	0.88	97.0%	85.7%
***RP4-605O3.4 RQ***	0.957	0.022	< 0.001*	0.915		1.000	0.74	92.4%	98.0%
Performance Characteristics in T2DM Patients Versus Healthy Control									
***TMEM173 RQ***	0.979	0.012	< 0.001*	0.956		1.000	4.005	93.9%	97.8%
***CHUK RQ***	0.981	0.014	< 0.001*	0.954		1.000	3.64	97.0%	97.8%
***hsa_miR_611 RQ***	0.986	0.009	< 0.001*	0.967		1.000	1.10	97.0%	95.6%
***hsa_miR_5192 RQ***	0.994	0.004	< 0.001*	0.985		1.000	0.0812	98.5%	95.6%
***hsa_miR_1976 RQ***	0.957	0.028	< 0.001*	0.903		1.000	0.695	98.5%	93.3%
***RP4-605O3.4 RQ***	0.993	0.006	< 0.001*	0.982		1.000	6.45	97.0%	97.8%
***AC074117.2 RQ***	0.916	0.033	< 0.001*	0.853		0.980	25.5	89.4%	84.4%

* p < 0.05, Significant.

**Figure 9:**
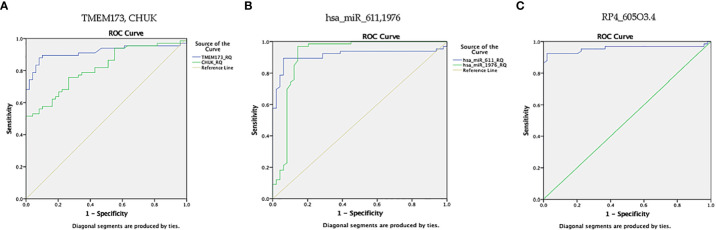
ROC curve represents the diagnostic accuracy of the investigated STING/NOD/IR RNA panel to discriminate between prediabetes group and T2DM group. **(A)** ROC curve analysis for serum *TMEM173* and *CHUK* mRNAs. **(B)** ROC curve analysis for serum *hsa-miR (-611* and *-1976)* miRNAs. **(C)** ROC curve analysis for serum *RP4-605O3.4* lncRNA.

ROC curve analysis was also performed for the investigated STING/NOD/IR RNA associated panel to discriminate prediabetes group and T2DM group and to determine the best cutoff values. For serum *TMEM173* and *CHUK* mRNAs the best cutoff values were ≥ 12.75 (AUC = 0.916) and ≥ 17.74 (AUC = 0.816), sensitivities were 89.4% and 75.8% specificities were 89.8% and 73.5% respectively. As regards serum *hsa-miR (-611* and *-1976* miRNAs the best cutoff values were ≥ 105.39 (AUC = 0.914) and ≥ 0.88 (AUC = 0.912), sensitivities were 89.4% and 97.0%, specificities were 93.9% and 85.7% respectively. And for serum *RP4-605O3.4* lncRNA the best cutoff value was ≤ 0.74 (AUC = 0.957), sensitivity was 92.4% specificity was 98.0%. These results suggest that the selected STING/NOD/IR RNA associated panel could be used as a tool to differentiate between prediabetes and type 2 diabetes cases (**Table 2** and **Figures 8**, **9**).

**Figure 10:**
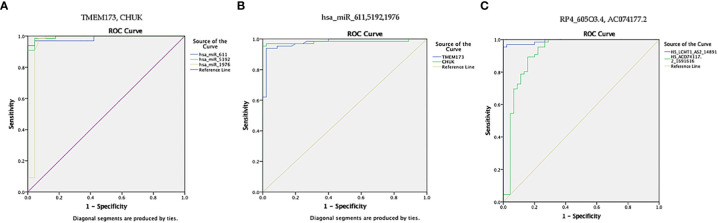
ROC curve presents the diagnostic accuracy of the investigated STING/NOD/IR RNA associated panel to discriminate between T2DM group and healthy controls. **(A)** ROC curve analysis for serum *TMEM173* and *CHUK* mRNAs. **(B)** ROC curve analysis for serum *hsa-miR (-611, -5192*, and *-1976)* mRNAs. **(C)** ROC curve analysis for serum *RP4-605O3.4* and *AC074117.2* lncRNAs.

Finally, ROC curve analysis was performed for the investigated STING/NOD/IR RNA associated panel to discriminate T2DM group and healthy controls. As for *TMEM173* and *CHUK* mRNAs the best cutoff values were ≥4.005 (AUC = 0.979), and ≥ 3.64 (AUC = 0.981), sensitivities were 93.9% and 97.0%, specificities were 97.8% and 97.8%, respectively. For serum *hsa-miR (-611, -5192*, and *-1976)* the best cutoff values were ≥1.10 (AUC = 0.986), ≥0.0812 (AUC = 0.994) and ≥0.695 (AUC = 0.957), sensitivities were 97.0%, 98.5%, and 98.5%, specificities were 95.6%, 95.6%, and 93.3%, respectively. While for serum *RP4-605O3.4* and *AC074117.2* lncRNAs the best cutoff values ≤6.45 (AUC = 0.993) and ≤25.5 (AUC = 0.916), sensitivities were 97.0% and 89.4%, specificities were 97.8% and 84.4% respectively. These findings suggest the potential use of this panel dysregulation is associated with T2DM pathogenesis (**Table 2** and **Figures 8**, **10**).

Patients with T2DM were subdivided into two groups according to their glycemic control. Good glycemic control was indicated by HbA1c <7%, while poor glycemic control was indicated by HbA1c ≥7% (42). There was clinical significance without statistically significant upregulation in the levels of expression of *hsa-miR-611* and clinically significant downregulation in the expression of *CHUK*, *hsa-miR (-611*, and *-5192)* and *RP4-605O3.4* in the individuals with poor glycemic control compared to individuals with good glycemic control (*p* = 0.040 and 0.010, respectively) suggesting possible use of these biomarkers together with HbA1c in discriminating glycemic control in T2DM patients. (**Table S5**).

The degree of insulin resistance was examined in relation to the expression of the insulin resistance associated panel. The prediabetic and T2DM groups were redivided into two groups; group which was insulin resistant (HOMA-IR ≥3.8) and the another was insulin sensitive (HOMA-IR <3.8) (43, 44). *TMEM173, hsa-miR (-611* and *-1976) and RP4-605O3.4* showed a significant difference in their expression (**Table S6**), which makes the use of these insulin resistance associated biomarkers together with HOMA-IR possible in differentiating insulin resistant from insulin sensitive patients. Multivariate analysis for prediction of insulin resistance has been used and showed that *miR-611* then *-1976* then *CHUK* were significant insulin resistance predictors beside HOMA-IR and HOMA-B (**Table 3**)

**Table 3:** Multivariate analysis for the predictors of IR.

Variable	*df*	Sig.	Score
**HOMA_IR**	66.987	1	<0.001**
**BMI**	19.646	1	<0.001**
**LDLc**	54.143	1	<0.001**
**HDL**	24.157	1	<0.001**
**TGs**	16.159	1	<0.001**
**HbA1c**	94.913	1	<0.001**
**Fasting Glucose**	75.420	1	<0.001**
**HOMA-B**	63.504	1	<0.001**
***TMEM173***	0.041	1	0.840
***CHUK***	6.241	1	0.012*
***hsa-miR-611***	7.702	1	0.006**
***hsa-miR-5192***	0.004	1	0.949
***has-miR-1976***	5.551	1	0.018*
***RP4-605O3.4***	1.930	1	0.165
***AC074117.2***	0.041	1	0.840

** p < 0.01, Highly Significant; * p < 0.05, Significant.

## 3 Discussion

Type 2 diabetes mellitus (T2DM) is a complex metabolic disease that poses a significant health burden. Early detection of the prediabetes stage is crucial to prevent complications from developing (45). However, studies on prediabetes and related biomarkers are limited. In this study, we aimed to more accurately determine those at risk of developing prediabetes and progressing to T2DM by combining several STING/NOD/IR RNA biomarkers. To achieve this, we explored the STimulator of Interferon Gene (STING)/Nucleotide-binding oligomerization domain (NOD)/insulin resistance (IR) signaling pathways and identified STING-related molecular target effectors. Our goal was to evaluate the diagnostic efficacy of this molecular RNA panel in the early detection and diagnosis of prediabetes and T2DM.

The cGAS-STING pathway is activated by host DNA that is aberrantly located in the cytosol, which contributes to enhanced inflammation-mediated insulin resistance (46). Overactivation of this system results in the generation of harmful pro-inflammatory cytokines. Consistent with these findings, knockout of cGAS-STING pathway downstream targets or pharmacological targeting of Ik-kinase alpha (IKKa) may improve glucose tolerance and insulin sensitivity in patients with T2DM (47, 48). *TMEM173*, commonly known as STING1, is a key adaptor protein found in the endoplasmic reticulum’s outer membrane. *TMEM173* was first recognized as a key molecule in innate immunity for its role in sensing invading DNA segments and inducing the production of interferon, as well as other pro-inflammatory cytokines *via* JAK/STAT signaling associated with the development of many diseases, including insulin resistance and diabetes (15). This is consistent with our results, which demonstrated an increase in the levels of expression of *TMEM173* mRNA in the prediabetic and T2DM groups.

The NOD signaling pathway acts as a key player in the inflammation induced and triggered by the cGAS-STING pathway. The CHUK gene is a crucial effector of the NLR pathway, and it is mapped to human Chr 10q24-q25. The encoded protein is a component of a cytokine-activated protein complex that acts as an inhibitor of the transcription factor NF-kappa-B signaling pathway, which is involved in the pathogenesis of prediabetes and T2DM (49). Our study found that the level of expression of CHUK mRNA increased progressively from the healthy control to the prediabetic and T2DM groups, indicating its role in the development of prediabetes and T2DM.

ncRNAs are particularly convincing candidates as diagnostic and prognostic biomarkers. Given the complexity of insulin resistance pathogenesis, a method based on the combination of both coding and non-coding biomarkers is preferable (28). Multiple studies have found that miRNAs play a crucial role in the molecular pathways that contribute to β-cell malfunction (50). Our candidate miRNAs *hsa-miR (-611, -5192*, and *-1976)* were first to be studied in this IR related study and found to be associated with prediabetes and T2DM. Some researchers suggested that miRNAs could be used as biomarkers for predicting susceptibility to diabetes (28). *Seyahn et al.*, found that assessing circulating levels of *miR126, miR148a* and *miR146a* miRNAs was useful in discriminating individuals with prediabetes from healthy controls, whereas *miR-34a* and *miR-30d* miRNAs levels were better in distinguishing T2DM patients (51).

Several lncRNAs are involved in different stages of insulin production and are linked to the development of insulin resistance (28). A study by *Sathishkumar and colleagues* suggested that levels of circulating lncRNAs in type 2 DM were correlated with different pathological factors, such as reduced glycemic control, broad inflammation, and cellular senescence (52). This is consistent with the findings by *Formichi et al.*, who discovered abnormal levels of some lncRNAs when comparing T2DM patients to healthy individuals. Surprisingly, these dysregulated lncRNAs were found to be positively related to insulin resistance and poor glucose tolerance (28). Remarkably, our study demonstrated that *RP4-605O3.4* and *AC074117.2* lncRNAs are differently expressed in the sera of T2DM patients, and their levels were significantly correlated with impaired glycemic control and insulin resistance.

Interactions between miRNAs and their target mRNA in 5′ UTR regions, coding-sequence, or gene promoters can regulate translation or activate transcription (53). Because lncRNAs containing miRNA response elements can compete with other RNA transcripts, they can theoretically operate as ceRNAs; and a single miRNA can control several target RNAs containing the unique miRNA response element, and these RNAs can be regulated by multiple miRNAs, laying the foundation for the establishment of a ceRNA network (54).

In this study, we hypothesized that molecular triggers, such as microbial dysbiosis, obesity, and DNA damage, lead to increased expression levels of *hsa-miR (-611, -5192, and -1976)* miRNAs. These miRNAs act as sponges, decreasing the expression of the *RP4-605O3.4* and *AC074117.2* lncRNAs, which act as ceRNAs, releasing and increasing levels of *TMEM173* and *CHUK* genes involved in the modulation of STING and NLR pathways, respectively, along with their downstream effectors. This leads to activation of the INF pathway, triggering low-grade inflammation and insulin resistance, eventually leading to prediabetes and T2DM.

The screened STING/NOD/IR RNA panel was found to be differently expressed in the studied groups. Our results demonstrated that the expression levels of *TMEM173* and *CHUK* mRNAs, together with *hsa-miR (-611, -5192* and *-1976)* miRNAs, progressively increased from the healthy control to the prediabetic group, reaching the highest levels in T2DM group. In contrast, the expression levels of *RP4-605O3.4* and *AC074117.2* lncRNAs progressively decreased from the healthy control to the prediabetic group, to reaching the lowest levels in the T2DM group. These findings raise the possibility of using this RNA panel as a circulating coding-noncoding biomarker panel for early detection of prediabetes and type 2 diabetes mellitus.

Furthermore, there was a significant upregulation in the expression of *hsa-miR-611* miRNA with significant downregulation in the expression of *RP4-605O3.4* lncRNA in the poor glycemic control group compared to individuals with good glycemic control. This suggests the possible use of these non-coding biomarkers, together with HbA1c, in discriminating glycemic control in T2DM patients. These findings are consistent with those of *Ortiz-Martínez M et al.*, who stated that novel serum biomarkers can be used with or even instead of conventional markers for detection and follow-up of diabetes (55). *TMEM173, CHUK* mRNAs, *hsa-miR (-611* and *-1976)* miRNAs, and *RP4-605O3.4* lncRNA were significantly useful in distinguishing insulin-resistant from insulin-sensitive groups, which matches the data collected by *Pielok et al.*, who revealed a set of ncRNAs that may act as potential predictors for hepatic insulin resistance (56).

This study had some limitations. The small sample size of the study and the limited number of genes may not represent the complete picture of the molecular changes occurring in individuals with prediabetes and T2DM. The use of bioinformatics analysis in selecting candidate mRNA genes related to type 2 DM and insulin resistance signaling has its advantages, but it is also subject to biases. One limitation is that this approach only considers gene expression at the transcriptional level, which may not fully capture the complexity of molecular changes associated with T2DM. To overcome this limitation, future studies may consider integrating data from different levels of molecular regulation, such as epigenetic and post-transcriptional modifications. This approach may provide a more comprehensive understanding of the molecular changes associated with T2DM. However, we plan to address these limitations in future research by conducting larger longitudinal studies with functional validation of the identified biomarkers through *in vitro* or *in vivo* experiments with more diverse populations to further explore the association between the identified biomarkers and the development and progression of type 2 diabetes.

In conclusion, this study identified novel STING/NOD/IR RNA panel (*TMEM173, CHUK* mRNAs*, hsa-miR (-611* and *-1976)* and *RP4-605O3.4* lncRNA) as pre-DM and T2DM associated biomarker panel. Our findings provide support for the association between this IR panel and other serum parameters in the progression of prediabetes to type 2 diabetes mellitus. However, prospective studies are needed to confirm these findings and explain the role of these genes in the risk of developing prediabetes and type 2 diabetes mellitus.

## 4 Materials and methods

### 4.1 Bioinformatics analysis

The identified STING/NOD/IR RNA associated panel was constructed through the following steps:

Retrieval of a set of candidate mRNA genes (*TMEM173* and *CHUK*) related to insulin resistance signaling pathways (STimulator of Interferon Gene and NLR pathways) from public microarray databases available at National Center of Biotechnology Information GEO (https://www.ncbi.nlm.nih.gov/geo/, accessed in May 2022), Gene atlas expression database; Verification of the expression of the retrieved candidate genes in skeletal muscles and adipose tissues *via* Gene Cards Human Genes database (https://www.genecards.org/), and *via* the Human protein atlas database (https://www.proteinatlas.org/) so as to decrease the false discovery rate; gene ontology was checked for TMEM173 and CHUK mRNAs using Enrichr (http://amp.pharm.mssm.edu/Enrichr) at p-value <0.05 which was defined to identify up-regulated and down-regulated genes in GO functional enrichments (**Supplementary Table S**7). Interactions of differently expressed biomarkers in IR and T2DM were analyzed *via* STRING database available at (https://string-db.org/). Identification of ncRNA (miRNA/lncRNA) genetic axis linked to the retrieved candidate mRNA genes in insulin resistance including *hsa-miR (-611, -5192* and *-1976)* miRNAs from miRWalk (http://mirwalk.umm.uni-heidelberg.de) and miRBASE databases available at (https://www.mirbase.org/). *RP4-605O3.4* and *AC074117.2* lncRNAs retrieved from DIANA Tools database available at (https://diana.e-ce.uth.gr/lncbasev3); these lncRNA–miRNA interactions were obtained through target gene prediction with confirmation of its expression in the interested tissues through NONCODE database and LNCipedia database available at (http://www.noncode.org/ and https://lncipedia.org/) respectively. Alignment between *hsa-miR-611* with *RP4-605O3.4* and *AC074117.2* lncRNAs through EMBL’s European Bioinformatics Institute database; Claustal omega database available at (https://www.ebi.ac.uk/Tools/msa/clustalo/), Detailed bioinformatics are illustrated in **Figure 1** and **Supplementary Figures S1–9**.

### 4.2 Study population

The current study was approved by the Ain Shams ethical committee, Faculty of Medicine. Participants were admitted to the Endocrinology Unit, Internal Medicine Department, Faculty of Medicine, Ain Shams University in the period between April 2022 and May 2022. There were 160 Egyptian unrelated participants. They were classified into 66 cases with type 2 diabetes mellitus, 49 were prediabetics with impaired glucose tolerance state, and there were 45 healthy volunteers came at the Ain Shams hospital outpatients’ clinics for routine checkups. A written informed consent was received from all the participants after being informed about the study. The diagnosis and classification of the diabetic and prediabetic state was made according to American Diabetes Association criteria 2022 for the diagnosis of type 2 diabetes mellitus and prediabetes (57).

Criteria for diagnosing diabetes and prediabetes was made based on the clinical presentation along with biochemical analyses, including fasting and 2 h post prandial blood glucose level, level of fasting insulin, glycated hemoglobin (HbA1c), lipid profile (total cholesterol, triglycerides, LDLc and HDLc), and albumin/creatinine ratio. HOMA-IR formula was calculated to assess the degree of tissue resistance to insulin. Upon hospital admission blood samples were collected and processed by centrifugation at 4000 rpm for 20 min. Resulting sera were kept in aliquots in a freezer at −80°C for further processing within 2 months.

### 4.3 Extraction of total RNA and quantitative real-time PCR

Total RNA was extracted and purified using miRNEasy extraction kit (Qiagen, Hilden, Germany) from the serum samples according to the manufacturer’s manual. Further assessments of RNA concentration, integrity and purity were performed using Nano-Drop instrument (Thermo Scientific, Waltham, MA, USA). miScript II RT Kit (Qiagen, Germany) was used for the preparation of cDNA libraries for mRNAs, lncRNAs and miRNAs. We added 4 µl of 5x miScript HiFlex Buffer, 2 µl of 10x miScript Nucleics Mix, 2 µl of miScript Reverse Transcriptase Mix and continued with RNase free water to 2 µg of extracted RNA to reach final volume of 20 µl, followed by incubation at 37°C for 60 minutes and at 95°C for 5 minutes using Rotor gene Thermal cycler (Thermo Electron, Waltham, MA, USA) then cDNA was stored at -20°C for further processing by real-time PCR.

The levels of the identified TMEM173 and CHUK mRNAs in serum were measured using QuantiTect SYBR Green PCR kit (Cat. no. 204143, Qiagen, Hilden, Germany) with specific primers for Hs_TMEM173_1_SG QuantiTect Primer Assay (NM_198282) and Hs_CHUK_1_SG QuantiTect Primer Assay (NM_001278) with 500 ng of cDNA added from the undiluted RT reaction to the individual PCR tube, alongside with Hs_GAPDH_1_SG QuantiTect Primer Assay (NM_002046) as the reference gene following the manufacturer’s protocol. Relative expression levels for *RP4-605O3.4* and *AC074117.2* lncRNAs were analyzed by QuantiNova Probe PCR Kit (Cat. no. 208252; Qiagen, Helman Germany) and QuantiNova LNA Probe PCR Assay for Human *RP4-605O3.4* (ENST00000548468) and *AC074117.2* (ENST00000417130) supplied by Qiagen. HS_HGDC_2467750 QuantiNova LNA PCR Probe (GeneGlobe ID: UPFH1126608) was used as Reference Assay following the manufacturer’s protocol.

The relative expression levels of *hsa-miR (-611, -5192* and *-1976)* miRNAs in serum were investigated by a miScript SYBRGreen PCR Kit (Cat. no. 218073; Qiagen, Helman Germany), a miScript universal primer and a miRNA‐specific forward primers of (Hs_miR-611 miScript Primer Assay) (Accession: MIMAT0003279) for *hsa-miR-611*, (Hs_miR-5192 miScript Primer Assay) (Accession: MIMAT0021123) for *hsa-miR-5192* and (Hs_miR-1976 miScript Primer Assay) (Accession: MIMAT0009451) for *hsa-miR-1976* with RNU‐6 was used as an internal reference control gene. All PCR primers were obtained from Qiagen, and the details of the primers used are supplied in **Supplementary Table S8**. The PCR program cycling conditions were adjusted according to the type of the measured RNA by the real time cycler Applied Biosystems 7500 (Cat. no. 4351105) according to the manufacturer’s protocol. Tenfold serial dilutions of cDNA (100 ng to 1 pg) were analyzed in duplicate to create a standard curve with at least five points using the PCR Kit with specific primers. We calculate the log of each sample dilution followed by getting the slope of the regression between the log values and the average Ct values. A PCR efficiency of 96% over six logs of template dilution was achieved, and as little as 1 pg of transcript was sensitively detected.

To ensure reproducibility of the assay, dilutions were prepared fresh and PCR reactions were carried out in triplicate experiments. The limit of detection was defined as the lowest dilution of cDNA in which all three replicates resulted in positive amplification. Threshold detection by using Applied Biosystems 7500 software v2.3 (51) that verify all the samples within geometric phase so delta Ct between any two samples remain constant. Applicants specifically were ensured by melting curve analysis. Negative non template control containing master mix and molecular grade water has been used to check any contamination. Melting curve was used to assess primer specificity. The 2−ΔΔCt technique was used to measure the expression of the IR specific RNA‐based candidate genes panel (58). genes were used as an internal control to normalize the raw data of the samples and compare these results to a reference sample. In this study, appropriate standardization strategies were carried out to recognize any experimental error introduced at any stage during extraction and processing of the RNA according to MIQE guidelines (59) (**Table S9**).

### 4.4 Statistical analysis

The software package of statistical analysis version 24 (SPSS24, IBM, Chicago, IL, USA) was used to statistically analyze the output data. Quantitative variables were analyzed using the median and the interquartile range (IQR) for the non-parametric data, and the mean ± SD and standard error were used for the raw symmetrically distributed numerical data. One-way ANOVAs and Mann–Whitney *U* test were also applied as appropriate. chi-Square tests were used to evaluate qualitative variables in number and percentage. Spearman correlation tests were applied to assess the correlations between quantitative variables. The Receiver Operating Characteristic (ROC) curve was used to evaluate the predictive value of the IR associated panel among investigated groups, in order to estimate the best cut-off points for different parameters with optimal sensitivities and specificities. Multivariate analysis was performed to determine the predictive power of different biomarkers for Insulin resistance and prediabetes. A p-value <0.05 was considered statistically significant.

## Data availability statement

The original contributions presented in the study are included in the article/**Supplementary material**. Further inquiries can be directed to the corresponding authors.

## Ethics statement

The studies involving human participants were reviewed and approved by Research Ethics Committee, Faculty of Medicine, Ain Shams University, Egypt, dated 21/7/2020, FWA 000017585. The patients/participants provided their written informed consent to participate in this study.

## Author contributions

HA shared in funding and supervision. MMe helped in resource acquisition. MH handled supervision, shared in the original draft reviewing. MMa shared in conceptualization, data creation, methodology, visualization, investigation, and reviewing. SA shared in the methodology and investigation. MK participated in formal analysis, validation, and original drafting. All authors contributed to the article and approved the submitted version.
