# Supplementary material for: Analysis of mRNA-miRNA-lncRNA differential expression in prediabetes/type 2 diabetes mellitus patients as potential players in insulin resistance
Source: Front Endocrinol (Lausanne). 2023 May 8;14:1131171. doi: 10.3389/fendo.2023.1131171 (PMC10200895; doi:10.3389/fendo.2023.1131171)
Supplement: Supplementary file 1 [file DataSheet_1.docx]

**Table S1.** The demographic, clinical and biochemical indices of the study groups.

|  | T2DM N = 66 | | | Impaired Glucose Tolerance N=49 | | | Healthy Control N = 45 | | | Test of Significance |
| --- | --- | --- | --- | --- | --- | --- | --- | --- | --- | --- |
| Sex  Male: n =56 (35 %)  Female: n =104 (65%) | 24 (36.4%)  42 (63.6%) | | | 14 (28.6%)  35 (71.4%) | | | 18 (40%)  27 (60%) | | | X^2^ = 1.439; p = 0.487 |
| Smoking  +ve: n = 80 (50%)  −ve: n = 80 (50%) | 34 (51.5%)  32 (48.5%) | | | 28 (57.1%)  21 (42.9%) | | | 18 (40%)  27 (60%) | | | X^2^ = 2.861; p = 0.239 |
| Family History of T2DM  +ve: n = 94 (58.8%)  −ve: n = 66 (41.3%) | 58 (87.9%)  8 (12.1%) | | | 27 (55.1%)  22 (44.9%) | | | 9 (20%)  36 (80%) | | | X^2^ = 51.259; p = <10¯3 * |
|  | Mean | ±SD | Standard error | Mean | ±SD | Standard error | Mean | ±SD | Standard error | Test of Significance |
| Age/years | 53.89 | 7.817 | 0.962 | 51.94 | 8.407 | 1.201 | 51.73 | 6.916 | 1.031 | F = 1.364; p = 0.259 |
| Duration of Diabetes/ years | 11.09 | 6.004 | 0.739 | NA | NA | NA | NA | NA | NA | NA |
| Fasting bl. Glucose (mg/dL) | 206.53 | 85.881 | 10.571 | 102.29 | 11.724 | 1.675 | 83.20 | 8.789 | 1.310 | F = 81.323; p = <10¯3 * |
| 2hr. PP bl. Glucose (mg/dL) | 298.86 | 123.947 | 15.257 | 142.06 | 34.011 | 4.859 | 112.36 | 15.812 | 2.357 | F = 85.113; p = <10¯3 * |
| HbA1c | 8.8311 | 2.33366 | 0.287 | 5.783 | 0.403 | 0.057 | 3.791 | 1.18030 | 0.175 | F = 132.455; p = <10¯3 * |
| Fasting Insulin (mIU/L) | 20.0126 | 9.61256 | 1.18322 | 15.364 | 5.872 | 0.838 | 3.943 | 2.695 | .40182 | F = 69.122; p = <10¯3 * |
| HOMA_IR | 9.0041 | 3.45002 | 0.424 | 3.819 | 1.356 | 0.193 | 0.809 | 0.551 | .08220 | F = 171.689; p =<10¯3 * |
| HOMA_B | 57.3030 | 12.72547 | 1.56640 | 107.551 | 30.513 | 4.359 | 199.177 | 20.810 | 3.10218 | F = 571.124; p =<10¯3 * |
| BMI (kg/m^2^) | 35.2030 | 5.29298 | 0.651 | 33.010 | 5.687 | 0.812 | 23.368 | 4.153 | .61923 | F = 75.551; p = <10¯3 * |
| Systolic Bl. Pr. (mmHg) | 138.03 | 15.213 | 1.873 | 130.61 | 17.279 | 2.468 | 117.67 | 8.160 | 1.216 | F = 27.019; p = <10¯3 * |
| Diastolic Bl. Pr. (mmHg) | 91.44 | 10.589 | 1.303 | 85.61 | 12.935 | 1.848 | 76.44 | 5.070 | .756 | F = 28.718; p = <10¯3 * |
| Lipid profile:  Total Cholesterol (mg/dL)  LDLc (mg/dL)  HDLc (mg/dL)  TGs (mg/dL) | 308.73  214.29  37.6515  244.7879 | 60.825  51.033  10.54294  91.32738 | 7.487  6.282  1.29775  11.24162 | 215.31  139.84  50.693  196.571 | 90.886  51.981  18.242  64.651 | 12.984  7.426  2.606  9.235 | 104.71  76.22  63.422  115.155 | 27.279  18.765  15.018  52.450 | 4.067  2.797  2.23889  7.81891 | F = 130.996; p =<10¯3 *  F = 130.214; p =<10¯3 *  F = 42.655; p =<10¯3 *  F = 40.919; p =<10¯3 * |
| Alb/Cr. Ratio (mg/g) | 231.9206 | 113.79017 | 14.00660 | 14.006 | 21.565 | 3.080 | 20.311 | 6.07462 | 0.905 | F = 154.124; p =<10¯3 * |

*F: One-way ANOVAs test, X2: chi-square test; * p < 0.05: Significant, p > 0.05: non-Significant. 2hr. PP bl. Glucose: 2hour Post Prandial blood Glucose, BMI:body mass index, HOMA_IR: Homeostatic Model Assessment for Insulin Resistance, LDLc: low density lipoprotein cholesterol, HDL: high density lipoprotein cholesterol, TGs: Triglycerides, Alb/Cr. Ratio: albumin/creatinine ratio.*

**Table S2.** Spearman Correlation analysis between the investigated STING/NOD/IR related RNA associated panel among prediabetic and the T2DM group (n=115).

|  | | *TMEM173 RQ* | *CHUK RQ* | *hsa_miR_611 RQ* | *hsa_miR_5192 RQ* | *hsa_miR_1976 RQ* | *RP4-605O3.4 RQ* | *AC074117.2 RQ* |
| --- | --- | --- | --- | --- | --- | --- | --- | --- |
| ***TMEM173 RQ*** | Correlation Coefficient | 1.000 | 0.390^**^ | 0.620^**^ | 0.041 | 0.425^**^ | -0.551^**^ | -0.174 |
|  | Sig. (2-tailed) | . | <10¯3 | <10¯3 | 0.665 | <10¯3 | <10¯3 | 0.063 |
| ***CHUK RQ*** | Correlation Coefficient | 0.390^**^ | 1.000 | 0.513^**^ | -0.065 | 0.393^**^ | -0.457^**^ | -0.037 |
|  | Sig. (2-tailed) | <10¯3 | . | <10¯3 | 0.493 | <10¯3 | <10¯3 | 0.692 |
| ***hsa_miR_611 RQ*** | Correlation Coefficient | 0.620^**^ | 0.513^**^ | 1.000 | 0.108 | 0.480^**^ | -0.529^**^ | -0.116 |
|  | Sig. (2-tailed) | <10¯3 | <10¯3 | . | 0.251 | <10¯3 | <10¯3 | 0.219 |
| ***hsa_miR_5192 RQ*** | Correlation Coefficient | 0.041 | -0.065 | 0.108 | 1.000 | 0.106 | -0.101 | -0.040 |
|  | Sig. (2-tailed) | 0.665 | 0.493 | 0.251 | . | 0.262 | 0.284 | 0.668 |
| ***hsa_miR_1976 RQ*** | Correlation Coefficient | 0.425^**^ | 0.393^**^ | 0.480^**^ | 0.106 | 1.000 | -0.563^**^ | 0.071 |
|  | Sig. (2-tailed) | <10¯3 | <10¯3 | <10¯3 | 0.262 | . | <10¯3 | 0.449 |
| ***RP4-605O3.4 RQ*** | Correlation Coefficient | -0.551^**^ | -0.457^**^ | -0.529^**^ | -0.101 | -0.563^**^ | 1.000 | 0.204^*^ |
|  | Sig. (2-tailed) | <10¯3 | <10¯3 | <10¯3 | 0.284 | <10¯3 | . | 0.029 |
| ***AC074117.2 RQ*** | Correlation Coefficient | -0.174 | -0.037 | -0.116 | -0.040 | 0.071 | 0.204^*^ | 1.000 |
|  | Sig. (2-tailed) | 0.063 | 0.692 | 0.219 | 0.668 | 0.449 | 0.029 | . |

Spearman correlation. *p* value, ** *p* < 0.01: Highly Significant, * *p* < 0.05: Significant, *p* > 0.05: non-Significant, RQ: Relative quantity (fold change) in gene expression.

**Table S3.** Spearman Correlation between the investigated IR predictor panel and the different clinicopathological factors among all the studied groups (n=160)

|  | | **TMEM173 RQ** | **CHUK RQ** | **hsa_miR_611 RQ** | **hsa_miR_5192 RQ** | **hsa_miR_1976 RQ** | **RP4-605O3.4 RQ** | **AC074117.2 RQ** | **Fasting bl. Glucose (mg/dL)** | **HbA1c** | **HOMA_IR** | **HOMA-B** | **BMI** | **Total Cholesterol (mg/dL)** | **LDLc (mg/dL)** | **Alb/Cr. Ratio**  **(mg/g)** |
| --- | --- | --- | --- | --- | --- | --- | --- | --- | --- | --- | --- | --- | --- | --- | --- | --- |
| **TMEM173 RQ** | **Correlation Coefficient** | 1.000 | 0.684^**^ | 0.793^**^ | 0.563^**^ | 0.634^**^ | -0.751^**^ | -0.524^**^ | 0.726^**^ | 0.771^**^ | 0.714^**^ | -0.762^**^ | 0.565^**^ | 0.624^**^ | 0.648^**^ | 0.583^**^ |
|  | **Sig. (2-tailed)** | . | <10¯3 | <10¯3 | <10¯3 | <10¯3 | <10¯3 | <10¯3 | <10¯3 | <10¯3 | <10¯3 | <10¯3 | <10¯3 | <10¯3 | <10¯3 | <10¯3 |
| **CHUK RQ** | **Correlation Coefficient** | 0.684^**^ | 1.000 | 0.755^**^ | 0.559^**^ | 0.654^**^ | -0.713^**^ | -0.467^**^ | 0.662^**^ | 0.754^**^ | 0.705^**^ | -0.708^**^ | 0.462^**^ | 0.551^**^ | 0.574^**^ | 0.493^**^ |
|  | **Sig. (2-tailed)** | <10¯3 | . | <10¯3 | <10¯3 | <10¯3 | <10¯3 | <10¯3 | <10¯3 | <10¯3 | <10¯3 | <10¯3 | <10¯3 | <10¯3 | <10¯3 | <10¯3 |
| **hsa_miR_611 RQ** | **Correlation Coefficient** | 0.793^**^ | 0.755^**^ | 1.000 | 0.626^**^ | 0.678^**^ | -0.755^**^ | -0.515^**^ | 0.708^**^ | 0.765^**^ | 0.814^**^ | -0.775^**^ | 0.539^**^ | 0.628^**^ | 0.695^**^ | 0.620^**^ |
|  | **Sig. (2-tailed)** | <10¯3 | <10¯3 | . | <10¯3 | <10¯3 | <10¯3 | <10¯3 | <10¯3 | <10¯3 | <10¯3 | <10¯3 | <10¯3 | <10¯3 | <10¯3 | <10¯3 |
| **hsa_miR_5192 RQ** | **Correlation Coefficient** | 0.563^**^ | 0.559^**^ | 0.626^**^ | 1.000 | 0.531^**^ | -.0590^**^ | -0.470^**^ | 0.547^**^ | 0.619^**^ | 0.643^**^ | -0.620^**^ | 0.513^**^ | 0.551^**^ | 0.507^**^ | 0.332^**^ |
|  | **Sig. (2-tailed)** | <10¯3 | <10¯3 | <10¯3 | . | <10¯3 | <10¯3 | <10¯3 | <10¯3 | <10¯3 | <10¯3 | <10¯3 | <10¯3 | <10¯3 | <10¯3 | <10¯3 |
| **hsa_miR_1976 RQ** | **Correlation Coefficient** | 0.634^**^ | 0.654^**^ | 0.678^**^ | 0.531^**^ | 1.000 | -0.665^**^ | -0.299^**^ | 0.647^**^ | 0.714^**^ | 0.716^**^ | -0.658^**^ | 0.479^**^ | 0.570^**^ | 0.620^**^ | 0.669^**^ |
|  | **Sig. (2-tailed)** | <10¯3 | <10¯3 | <10¯3 | <10¯3 | . | <10¯3 | <10¯3 | <10¯3 | <10¯3 | <10¯3 | <10¯3 | <10¯3 | <10¯3 | <10¯3 | <10¯3 |
| **RP4-605O3.4 RQ** | **Correlation Coefficient** | -0.751^**^ | -0.713^**^ | -0.755^**^ | -0.590^**^ | -0.665^**^ | 1.000 | 0.519^**^ | -0.760^**^ | -0.839^**^ | -0.783^**^ | 0.787^**^ | -0.539^**^ | -0.631^**^ | -0.680^**^ | -0.603^**^ |
|  | **Sig. (2-tailed)** | <10¯3 | <10¯3 | <10¯3 | <10¯3 | <10¯3 | . | <10¯3 | <10¯3 | <10¯3 | <10¯3 | <10¯3 | <10¯3 | <10¯3 | <10¯3 | <10¯3 |
| **AC074117.2 RQ** | **Correlation Coefficient** | -0.524^**^ | -0.467^**^ | -0.515^**^ | -0.470^**^ | -0.299^**^ | 0.519^**^ | 1.000 | -0.485^**^ | -0.490^**^ | -0.477^**^ | 0.589^**^ | -0.379^**^ | -0.390^**^ | -0.409^**^ | -0.362^**^ |
|  | **Sig. (2-tailed)** | <10¯3 | <10¯3 | <10¯3 | <10¯3 | <10¯3 | <10¯3 | . | <10¯3 | <10¯3 | <10¯3 | <10¯3 | <10¯3 | <10¯3 | <10¯3 | <10¯3 |
| **Fasting bl. Glucose (mg/dL)** | **Correlation Coefficient** | 0.726^**^ | 0.662^**^ | 0.708^**^ | 0.547^**^ | 0.647^**^ | -0.760^**^ | -0.485^**^ | 1.000 | 0.839^**^ | 0.805^**^ | -0.808^**^ | 0.548^**^ | 0.659^**^ | 0.730^**^ | 0.647^**^ |
|  | **Sig. (2-tailed)** | <10¯3 | <10¯3 | <10¯3 | <10¯3 | <10¯3 | <10¯3 | <10¯3 | . | <10¯3 | <10¯3 | <10¯3 | <10¯3 | <10¯3 | <10¯3 | <10¯3 |
| **HbA1c** | **Correlation Coefficient** | 0.771^**^ | 0.754^**^ | 0.765^**^ | 0.619^**^ | 0.714^**^ | -0.839^**^ | -0.490^**^ | 0.839^**^ | 1.000 | 0.859^**^ | -0.845^**^ | 0.584^**^ | 0.705^**^ | 0.754^**^ | 0.662^**^ |
|  | **Sig. (2-tailed)** | <10¯3 | <10¯3 | <10¯3 | <10¯3 | <10¯3 | <10¯3 | <10¯3 | <10¯3 | . | <10¯3 | <10¯3 | <10¯3 | <10¯3 | <10¯3 | <10¯3 |
| **HOMA_IR** | **Correlation Coefficient** | 0.714^**^ | 0.705^**^ | 0.814^**^ | 0.643^**^ | 0.716^**^ | -0.783^**^ | -0.477^**^ | 0.805^**^ | 0.859^**^ | 1.000 | -0.821^**^ | 0.587^**^ | 0.694^**^ | 0.759^**^ | 0.657^**^ |
|  | **Sig. (2-tailed)** | <10¯3 | <10¯3 | <10¯3 | <10¯3 | <10¯3 | <10¯3 | <10¯3 | <10¯3 | <10¯3 | . | <10¯3 | <10¯3 | <10¯3 | <10¯3 | <10¯3 |
| **HOMA-B** | **Correlation Coefficient** | -0.762^**^ | -.0708^**^ | -0.775^**^ | -0.620^**^ | -.0658^**^ | 0.787^**^ | 0.589^**^ | -0.808^**^ | -0.845^**^ | -0.821^**^ | 1.000 | -0.487^**^ | -0.640^**^ | -0.704^**^ | -0.670^**^ |
|  | **Sig. (2-tailed)** | <10¯3 | <10¯3 | <10¯3 | <10¯3 | <10¯3 | <10¯3 | <10¯3 | <10¯3 | <10¯3 | <10¯3 | . | <10¯3 | <10¯3 | <10¯3 | <10¯3 |
| **BMI**  **(kg/m^2^)** | **Correlation Coefficient** | 0.565^**^ | 0.462^**^ | 0.539^**^ | 0.513^**^ | 0.479^**^ | -0.539^**^ | -0.379^**^ | 0.548^**^ | 0.584^**^ | 0.587^**^ | -0.487^**^ | 1.000 | 0.768^**^ | 0.727^**^ | .0386^**^ |
|  | **Sig. (2-tailed)** | <10¯3 | <10¯3 | <10¯3 | <10¯3 | <10¯3 | <10¯3 | <10¯3 | <10¯3 | <10¯3 | <10¯3 | <10¯3 | . | <10¯3 | <10¯3 | <10¯3 |
| **Total Cholesterol (mg/dL)** | **Correlation Coefficient** | 0.624^**^ | 0.551^**^ | 0.628^**^ | 0.551^**^ | 0.570^**^ | -0.631^**^ | -0.390^**^ | 0.659^**^ | 0.705^**^ | 0.694^**^ | -0.640^**^ | 0.768^**^ | 1.000 | 0.880^**^ | 0.499^**^ |
|  | **Sig. (2-tailed)** | <10¯3 | <10¯3 | <10¯3 | <10¯3 | <10¯3 | <10¯3 | <10¯3 | <10¯3 | <10¯3 | <10¯3 | <10¯3 | <10¯3 | . | <10¯3 | <10¯3 |
| **LDLc (mg/dL)** | **Correlation Coefficient** | 0.648^**^ | 0.574^**^ | 0.695^**^ | 0.507^**^ | 0.620^**^ | -0.680^**^ | -0.409^*^ | 0.730^**^ | 0.754^**^ | 0.759^**^ | -0.704^**^ | 0.727^**^ | 0.880^**^ | 1.000 | 0.601^**^ |
|  | **Sig. (2-tailed)** | <10¯3 | <10¯3 | <10¯3 | <10¯3 | <10¯3 | <10¯3 | <10¯3 | <10¯3 | <10¯3 | <10¯3 | <10¯3 | <10¯3 | <10¯3 | . | <10¯3 |
| **Alb/Cr. Ratio**  **(mg/g)** | **Correlation Coefficient** | 0.583^**^ | 0.493^**^ | 0.620^**^ | 0.332^**^ | 0.669^**^ | -0.603^**^ | -0.362^**^ | 0.647^**^ | 0.662^**^ | 0.657^**^ | -0.670^**^ | 0.386^**^ | 0.499^**^ | 0.601^**^ | 1.000 |
|  | **Sig. (2-tailed)** | <10¯3 | <10¯3 | <10¯3 | <10¯3 | <10¯3 | <10¯3 | <10¯3 | <10¯3 | <10¯3 | <10¯3 | <10¯3 | <10¯3 | <10¯3 | <10¯3 | . |

Spearman correlation. p value, ** p < 0.01: Highly Significant, * p < 0.05: Significant, p > 0.05: non-Significant, RQ: Relative quantity (fold change) in gene expression.

**Table S4**. Spearman Correlation analysis between the investigated IR predictor panel among prediabetic and the healthy groups (n=94)

|  | | **TMEM173 RQ** | **CHUK RQ** | **hsa_miR_611 RQ** | **hsa_miR_5192 RQ** | **hsa_miR_1976 RQ** | **RP4-605O3.4 RQ** | **AC074117.2 RQ** |
| --- | --- | --- | --- | --- | --- | --- | --- | --- |
| **TMEM173 RQ** | **Correlation Coefficient** | 1.000 | 0.628^**^ | 0.697^**^ | 0.603^**^ | 0.387^**^ | -0.592^**^ | -0.481^**^ |
|  | **Sig. (2-tailed)** | . | <10¯3 | <10¯3 | <10¯3 | <10¯3 | <10¯3 | <10¯3 |
| **CHUK RQ** | **Correlation Coefficient** | 0.628^**^ | 1.000 | 0.680^**^ | 0.744^**^ | 0.543^**^ | -0.546^**^ | -0.469^**^ |
|  | **Sig. (2-tailed)** | <10¯3 | . | <10¯3 | <10¯3 | <10¯3 | <10¯3 | <10¯3 |
| **hsa_miR_611 RQ** | **Correlation Coefficient** | 0.697^**^ | 0.680^**^ | 1.000 | 0.781^**^ | 0.514^**^ | -0.612^**^ | -0.562^**^ |
|  | **Sig. (2-tailed)** | <10¯3 | <10¯3 | . | <10¯3 | <10¯3 | <10¯3 | <10¯3 |
| **hsa_miR_5192 RQ** | **Correlation Coefficient** | 0.603^**^ | 0.744^**^ | 0.781^**^ | 1.000 | 0.545^**^ | -0.605^**^ | -0.498^**^ |
|  | **Sig. (2-tailed)** | <10¯3 | <10¯3 | <10¯3 | . | <10¯3 | <10¯3 | <10¯3 |
| **hsa_miR_1976 RQ** | **Correlation Coefficient** | 0.387^**^ | 0.543^**^ | 0.514^**^ | 0.545^**^ | 1.000 | -0.243^*^ | -0.130 |
|  | **Sig. (2-tailed)** | <10¯3 | <10¯3 | <10¯3 | <10¯3 | . | 0.018 | 0.213 |
| **RP4-605O3.4 RQ** | **Correlation Coefficient** | -0.592^**^ | -0.546^**^ | -0.612^**^ | -0.605^**^ | -0.243^*^ | 1.000 | 0.460^**^ |
|  | **Sig. (2-tailed)** | <10¯3 | <10¯3 | <10¯3 | <10¯3 | 0.018 | . | <10¯3 |
| **AC074117.2 RQ** | **Correlation Coefficient** | -0.481^**^ | -0.469^**^ | -0.562^**^ | -0.498^**^ | -0.130 | 0.460^**^ | 1.000 |
|  | **Sig. (2-tailed)** | <10¯3 | <10¯3 | <10¯3 | <10¯3 | 0.213 | <10¯3 | . |

Spearman correlation. p value, ** p < 0.01: Highly Significant, * p < 0.05: Significant, p > 0.05: non-Significant, RQ: Relative quantity (fold change) in gene expression.

**Table S5.** Relation between the expression of the Insulin resistance predictor panel and the glycemic control in the T2DM group.

|  | **Poor Glycemic Control HbA1c ≥ 7**  **no.=52** | | **Good Glycemic Control HbA1c < 7**  **no.=14** | | **U** | ***p*** |
| --- | --- | --- | --- | --- | --- | --- |
|  | **Median** | **IQR** | **Median** | **IQR** |  |  |
| ***TMEM173 RQ*** | *77.500* | 17.51 - 282.5 | 66.525 | 0.25 - 7.39 | 352.000 | 0.851 |
| ***CHUK RQ*** | 93.464 | 17.79 - 601.83 | 230.875 | 0.49 - 16.98 | 328.000 | 0.572 |
| ***hsa_miR_611 RQ*** | 382.767 | 199.3 - 1169.27 | 733.254 | 0.42 - 44.44 | 233.000 | 0.040 |
| ***hsa_miR_5192 RQ*** | 14.041 | 2.89 - 165.33 | 39.560 | 0.01 - 23.24 | 329.000 | 0.583 |
| ***hsa_miR_1976 RQ*** | 2.506 | 1.266 - 4.3215 | 3.066 | 0.008 - 0.735 | 303.500 | 0.343 |
| ***RP4-605O3.4 RQ*** | .006 | 0.003 - 0.070 | 0.085 | 4.23 - 652 | 200.000 | 0.010 |
| ***AC074117.2 RQ*** | 1.500 | 0.5 – 10.0 | 1.460 | 1.35 - 338.84 | 345.500 | 0.771 |

Mann–Whitney test: After Bonferroni correction (* p < 0.007: Significant, p > 0.007: non-Significant).

**Table S6.** Relation between the expression of the Insulin resistance predictor panel and the insulin resistance in both Prediabetic and T2DM groups.

|  | **Insulin resistant ≥ 3.8**  **no.=86** | | **Insulin sensitive < 3.8**  **no.=29** | | **U** | ***p*** |
| --- | --- | --- | --- | --- | --- | --- |
|  | **Median** | **IQR** | **Median** | **IQR** |  |  |
| ***TMEM173 RQ*** | 23.025 | 7.6 - 134 | 4.300 | 0.06 - 3.5 | 564.500 | < 0.001* |
| ***CHUK RQ*** | 24.255 | 13 - 345 | 13.828 | 0.21 - 12.4 | 839.500 | 0.009 |
| ***hsa_miR_611 RQ*** | 335.470 | 48.4 - 783.13 | 24.433 | 0.18 - 19.37 | 489.000 | < 0.001* |
| ***hsa_miR_5192 RQ*** | 17.371 | 2.74 - 126.24 | 14.978 | 0.002 - 4.76 | 1100.000 | 0.344 |
| ***hsa_miR_1976 RQ*** | 2.001 | 0.89 - 4.040 | .250 | 0.006 - 0.44 | 565.500 | < 0.001* |
| ***RP4-605O3.4 RQ*** | .070 | 0.004 - 2.900 | 5.200 | 12.0 – 1700.0 | 471.000 | < 0.001* |
| ***AC074117.2 RQ*** | 2.255 | 0.5 – 14.0 | 10.000 | 8.6 – 357.0 | 1067.500 | 0.247 |

Mann–Whitney test: *p* value, After Bonferroni correction (* p < 0.007: Significant, p > 0.007: non-Significant

**Table S7.** Gene ontology& pathway enrichment analysis

| **Index** | **Name** | **P-value** | **Adjusted p-value** | **Odds Ratio** | **Combined score** |
| --- | --- | --- | --- | --- | --- |
| 1 | I-kappaB phosphorylation (GO:0007252) | 0.003495 | 0.07696 | 370.07 | 2093.27 |
| 2 | positive regulation of interferon-alpha production (GO:0032727) | 0.006980 | 0.07696 | 175.21 | 869.87 |
| 3 | regulation of interferon-alpha production (GO:0032647) | 0.008718 | 0.07696 | 138.67 | 657.63 |
| 4 | cellular response to cadmium ion (GO:0071276) | 0.009413 | 0.07696 | 127.99 | 597.17 |
| 5 | stress-activated protein kinase signaling cascade (GO:0031098) | 0.009413 | 0.07696 | 127.99 | 597.17 |
| 6 | response to cadmium ion (GO:0046686) | 0.01011 | 0.07696 | 118.84 | 546.01 |
| 7 | TRIF-dependent toll-like receptor signaling pathway (GO:0035666) | 0.01045 | 0.07696 | 114.74 | 523.28 |
| 8 | MyD88-independent toll-like receptor signaling pathway (GO:0002756) | 0.01080 | 0.07696 | 110.91 | 502.19 |
| 9 | cellular response to virus (GO:0098586) | 0.01219 | 0.07719 | 97.84 | 431.21 |
| 10 | regulation of tumor necrosis factor-mediated signaling pathway (GO:0010803) | 0.02013 | 0.07789 | 58.29 | 227.67 |
| 11 | response to reactive oxygen species (GO:0000302) | 0.02047 | 0.07789 | 57.28 | 222.76 |
| 12 | I-kappaB kinase/NF-kappaB signaling (GO:0007249) | 0.02150 | 0.07789 | 54.46 | 209.10 |
| 13 | stress-activated MAPK cascade (GO:0051403) | 0.02150 | 0.07789 | 54.46 | 209.10 |
| 14 | cellular response to reactive oxygen species (GO:0034614) | 0.02185 | 0.07789 | 53.58 | 204.87 |
| 15 | antigen processing and presentation of exogenous peptide antigen via MHC class I, TAP-dependent (GO:0002479) | 0.02528 | 0.07789 | 46.11 | 169.60 |
| 16 | regulation of cytokine-mediated signaling pathway (GO:0001959) | 0.02562 | 0.07789 | 45.48 | 166.66 |
| 17 | NIK/NF-kappaB signaling (GO:0038061) | 0.02562 | 0.07789 | 45.48 | 166.66 |
| 18 | positive regulation of type I interferon production (GO:0032481) | 0.02664 | 0.07789 | 43.68 | 158.34 |
| 19 | antigen processing and presentation of exogenous peptide antigen via MHC class I (GO:0042590) | 0.02699 | 0.07789 | 43.11 | 155.72 |
| 20 | negative regulation of NF-kappaB transcription factor activity (GO:0032088) | 0.02733 | 0.07789 | 42.55 | 153.19 |
| 21 | toll-like receptor signaling pathway (GO:0002224) | 0.03142 | 0.08043 | 36.86 | 127.53 |
| 22 | interleukin-1-mediated signaling pathway (GO:0070498) | 0.03244 | 0.08043 | 35.66 | 122.26 |
| 23 | response to tumor necrosis factor (GO:0034612) | 0.03788 | 0.08043 | 30.40 | 99.52 |
| 24 | Fc-epsilon receptor signaling pathway (GO:0038095) | 0.03821 | 0.08043 | 30.13 | 98.35 |
| 25 | Fc receptor signaling pathway (GO:0038093) | 0.03855 | 0.08043 | 29.85 | 97.19 |
| 26 | cellular response to lectin (GO:1990858) | 0.03957 | 0.08043 | 29.06 | 93.87 |
| 27 | stimulatory C-type lectin receptor signaling pathway (GO:0002223) | 0.03957 | 0.08043 | 29.06 | 93.87 |
| 28 | tumor necrosis factor-mediated signaling pathway (GO:0033209) | 0.03991 | 0.08043 | 28.81 | 92.80 |
| 29 | innate immune response activating cell surface receptor signaling pathway (GO:0002220) | 0.04092 | 0.08043 | 28.07 | 89.72 |
| 30 | cellular response to oxidative stress (GO:0034599) | 0.04294 | 0.08159 | 26.71 | 84.07 |
| 31 | cellular response to metal ion (GO:0071248) | 0.04497 | 0.08268 | 25.47 | 78.99 |
| 32 | negative regulation of DNA-binding transcription factor activity (GO:0043433) | 0.04900 | 0.08552 | 23.30 | 70.27 |
| 33 | cellular response to interleukin-1 (GO:0071347) | 0.05301 | 0.08552 | 21.47 | 63.06 |
| 34 | positive regulation of NF-kappaB transcription factor activity (GO:0051092) | 0.05301 | 0.08552 | 21.47 | 63.06 |
| 35 | peptidyl-serine phosphorylation (GO:0018105) | 0.05335 | 0.08552 | 21.33 | 62.52 |
| 36 | T cell receptor signaling pathway (GO:0050852) | 0.05401 | 0.08552 | 21.06 | 61.46 |
| 37 | peptidyl-serine modification (GO:0018209) | 0.05768 | 0.08752 | 19.67 | 56.11 |
| 38 | positive regulation of I-kappaB kinase/NF-kappaB signaling (GO:0043123) | 0.05834 | 0.08752 | 19.43 | 55.22 |
| 39 | antigen receptor-mediated signaling pathway (GO:0050851) | 0.06299 | 0.09206 | 17.94 | 49.61 |
| 40 | cellular response to tumor necrosis factor (GO:0071356) | 0.06597 | 0.09400 | 17.10 | 46.48 |
| 41 | regulation of I-kappaB kinase/NF-kappaB signaling (GO:0043122) | 0.07583 | 0.1054 | 14.78 | 38.11 |
| 42 | inflammatory response (GO:0006954) | 0.07779 | 0.1056 | 14.38 | 36.73 |
| 43 | positive regulation of DNA-binding transcription factor activity (GO:0051091) | 0.08300 | 0.1100 | 13.43 | 33.44 |
| 44 | innate immune response (GO:0045087) | 0.1010 | 0.1284 | 10.90 | 24.99 |
| 45 | MAPK cascade (GO:0000165) | 0.1014 | 0.1284 | 10.87 | 24.87 |
| 46 | cellular response to oxygen-containing compound (GO:1901701) | 0.1077 | 0.1335 | 10.18 | 22.69 |
| 47 | phosphorylation (GO:0016310) | 0.1319 | 0.1600 | 8.18 | 16.58 |
| 48 | cellular response to cytokine stimulus (GO:0071345) | 0.1570 | 0.1864 | 6.76 | 12.52 |
| 49 | protein phosphorylation (GO:0006468) | 0.1612 | 0.1875 | 6.56 | 11.98 |
| 50 | positive regulation of nucleic acid-templated transcription (GO:1903508) | 0.1657 | 0.1889 | 6.37 | 11.44 |
| 51 | positive regulation of intracellular signal transduction (GO:1902533) | 0.1762 | 0.1969 | 5.95 | 10.33 |
| 52 | cytokine-mediated signaling pathway (GO:0019221) | 0.1981 | 0.2172 | 5.21 | 8.43 |
| 53 | positive regulation of transcription by RNA polymerase II (GO:0045944) | 0.2777 | 0.2986 | 3.51 | 4.49 |
| 54 | cellular protein modification process (GO:0006464) | 0.3081 | 0.3252 | 3.09 | 3.63 |
| 55 | positive regulation of transcription, DNA-templated (GO:0045893) | 0.3474 | 0.3601 | 2.65 | 2.80 |
| 56 | regulation of transcription by RNA polymerase II (GO:0006357) | 0.5588 | 0.5653 | 1.34 | 0.78 |
| 57 | regulation of transcription, DNA-templated (GO:0006355) | 0.5653 | 0.5653 | 1.32 | 0.75 |

**Table S8:** Primers used for qPCR

| **Primer name** | **Gene ID** | **Cat.no.** | **Tm** | **Product size** | **Program setup and no. of cycles** |
| --- | --- | --- | --- | --- | --- |
| ***TMEM173 RQ*** | NM_198282 | 249900  **Gene Globe ID:** QT00055440 | 70 °C | 125 bp | **PCR initial activation step**: 15 min at 95°C  **3-step cycling:**  **Denaturation**; 15 s at 94°C  **Annealing**; 30 s at 55°C  **Extension**; 34 s 72°C  **Number of cycles**: 40 cycles |
| ***CHUK RQ*** | NM_001278 | 249900  **Gene Globe ID:** QT00040614 | 70 °C | 100 bp |  |
| ***GAPDH*** | NM_002046 | 249900  **Gene Globe ID:** QT00079247 | 70°C | 95 bp |  |
| ***hsa_miR_611*** | MIMAT0003279 | 339350  **Gene Globe ID:** ZP00001890 | 75 °C | 85 bp | **PCR initial activation step**: 15 min at 95°C  **3-step cycling:**  **Denaturation**; 15 s at 94°C  **Annealing**; 30 s at 55°C  **Extension**; 34 s 70°C  **Number of cycles**: 40 cycles |
| ***hsa_miR_5192*** | MIMAT0021123 | 339350  **Gene Globe ID:** ZP00001600 | 75 °C | 85 bp |  |
| ***hsa_miR_1976*** | MIMAT0009451 | 339350  **Gene Globe ID:** ZP00000388 | 75 °C | 85 bp |  |
| ***RP4-605O3.4*** | ENST00000548468 | **Gene Globe ID:** SBH0573696 | 58.8 °C | 108 bp | **PCR initial activation step**: 2 min at 95°C  **2-step cycling:**  **Denaturation**; 5 s at 95°C  **Combined annealing / extension**; 10 s at 60°C  **Number of cycles**: 40 cycles |
| ***AC074117.2*** | ENST00000417130 | **Gene Globe ID:** UPFH0284997 | 58.8 °C | 82 bp |  |

**Table S9.** MIQE guidelines checklist:

| **ITEM TO CHECK** | **CHECKLIST** |
| --- | --- |
| **EXPERIMENTAL DESIGN** |  |
| Definition of experimental and control groups | **done** |
| Number within each group | **done** |
| Acknowledgement of authors' contributions | **done** |
| Description | **done** |
| Processing procedure | **done** |
| If frozen - how and how quickly? | **done** |
| Sample storage conditions and duration (especially for FFPE samples) | **done** |
| Procedure and/or instrumentation | **done** |
| Name of kit and details of any modifications | **done** |
| Details of DNase or RNAse treatment | **done (acc.to manufactuer protocol)** |
| Contamination assessment (DNA or RNA) | **done** |
| Nucleic acid quantification | **done** |
| Instrument and method | **done** |
| Purity (A260/A280) | **done** |
| Yield | **done** |
| RNA integrity method/instrument | **done** |
| **REVERSE TRANSCRIPTION** |  |
| Complete reaction conditions | **done** |
| Amount of RNA and reaction volume | **done** |
| Priming oligonucleotide (if using GSP) and concentration | **done** |
| Reverse transcriptase and concentration | **done** |
| Temperature and time | **done** |
| Manufacturer of reagents and catalogue numbers | **done** |
| Storage conditions of cDNA | **done** |
| **qPCR TARGET INFORMATION** |  |
| Sequence accession number | **done** |
| Amplicon length | **done** |
| *In silico* specificity screen (BLAST, etc) |  |
| **qPCR OLIGONUCLEOTIDES** |  |
| Primer sequences | **done** |
| Location and identity of any modifications | done |
| Manufacturer of oligonucleotides | done |
| **qPCR PROTOCOL** |  |
| Complete reaction conditions | **done (acc.to manufactuer protocol)** |
| Reaction volume and amount of cDNA/DNA | **done** |
| Primer, (probe), Mg++ and dNTP concentrations | **done (acc.to manufactuer protocol)** |
| Polymerase identity and concentration | **done (acc.to manufactuer protocol)** |
| Buffer/kit identity and manufacturer | **done (acc.to manufactuer protocol)** |
| Exact chemical constitution of the buffer | **done (acc.to manufactuer protocol)** |
| Additives (SYBR Green I, DMSO, etc.) | **done (acc.to manufactuer protocol)** |
| Complete thermocycling parameters | **done (acc.to manufactuer protocol)** |
| Reaction setup (manual/robotic) | **done (acc.to manufactuer protocol)** |
| Manufacturer of qPCR instrument | **done** |
| **qPCR VALIDATION** |  |
| Specificity (gel, sequence, melt, or digest) | **done** |
| For SYBR Green I, Cq of the NTC | **done** |
| Standard curves with slope and y-intercept | **Done by software** |
| PCR efficiency calculated from slope | **Done by software** |
| r2 of standard curve | **Done by software** |
| Linear dynamic range | **Done by software** |
| Cq variation at lower limit | **Done by software** |
| Evidence for limit of detection | **done** |
| **DATA ANALYSIS** |  |
| qPCR analysis program (source, version) | **done** |
| Cq method determination | **done** |
| Outlier identification and disposition | **done** |
| Results of NTCs | **done** |
| Justification of number and choice of reference genes | **done** |
| Description of normalisation method | **done** |
| Number and concordance of biological replicates | **done** |
| Number and stage (RT or qPCR) of technical replicates | **done** |
| Repeatability (intra-assay variation) | **done** |
| Statistical methods for result significance | **Done** |
| Software (source, version) | **Done** |

A: KEGG: Kyoto Encyclopedia of Genes and Genomes database


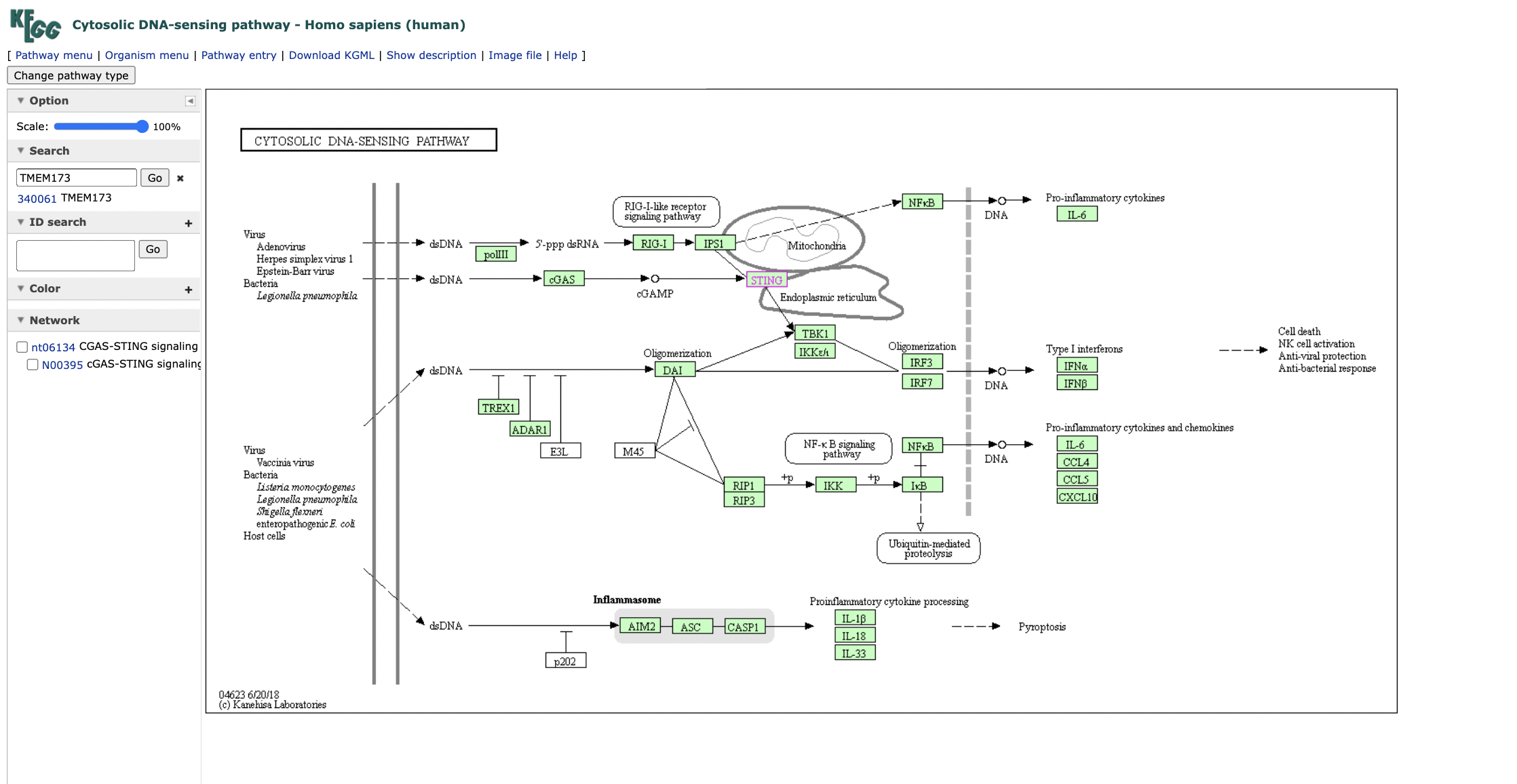


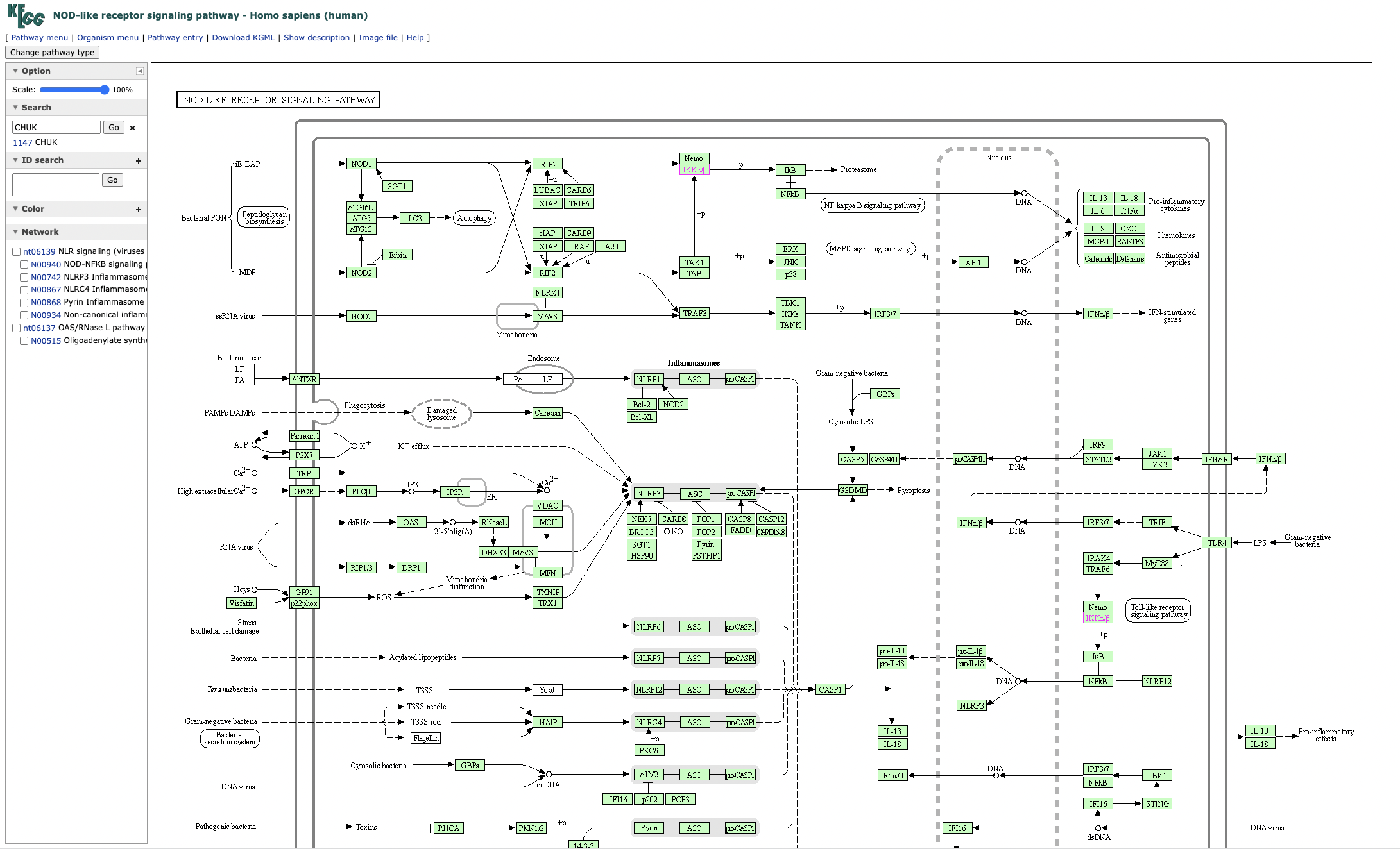


B: **National Center of Biotechnology Information GEO**


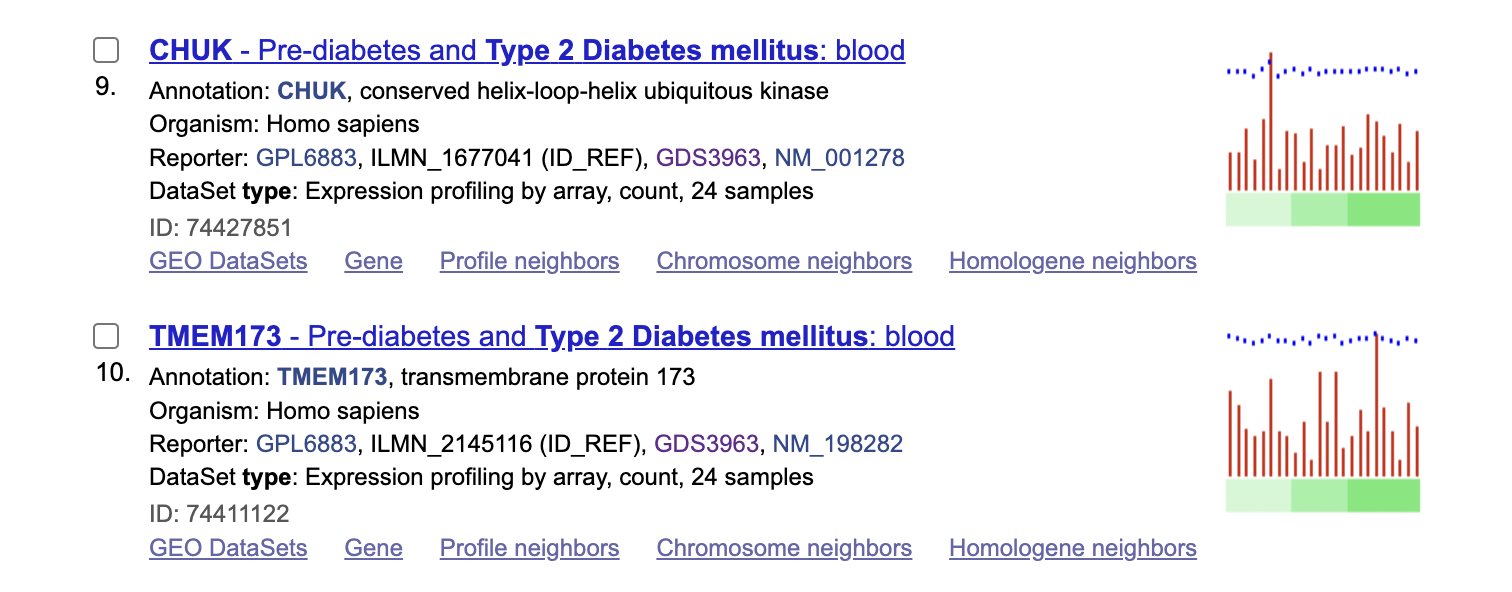


**Figure S1: (A) Retrieval** of a candidate genes (*TMEM173 & CHUK* mRNAs) related to insulin resistance signaling pathways (STimulator of Interferon Gene and NLR signaling pathways) from public microarray databases available at KEGG: Kyoto Encyclopedia of Genes and Genomes (<https://www.genome.jp/kegg/>) and **(B)** **National Center of Biotechnology Information GEO (https://www.ncbi.nlm.nih.gov/geo/**); accessed on May 2022.


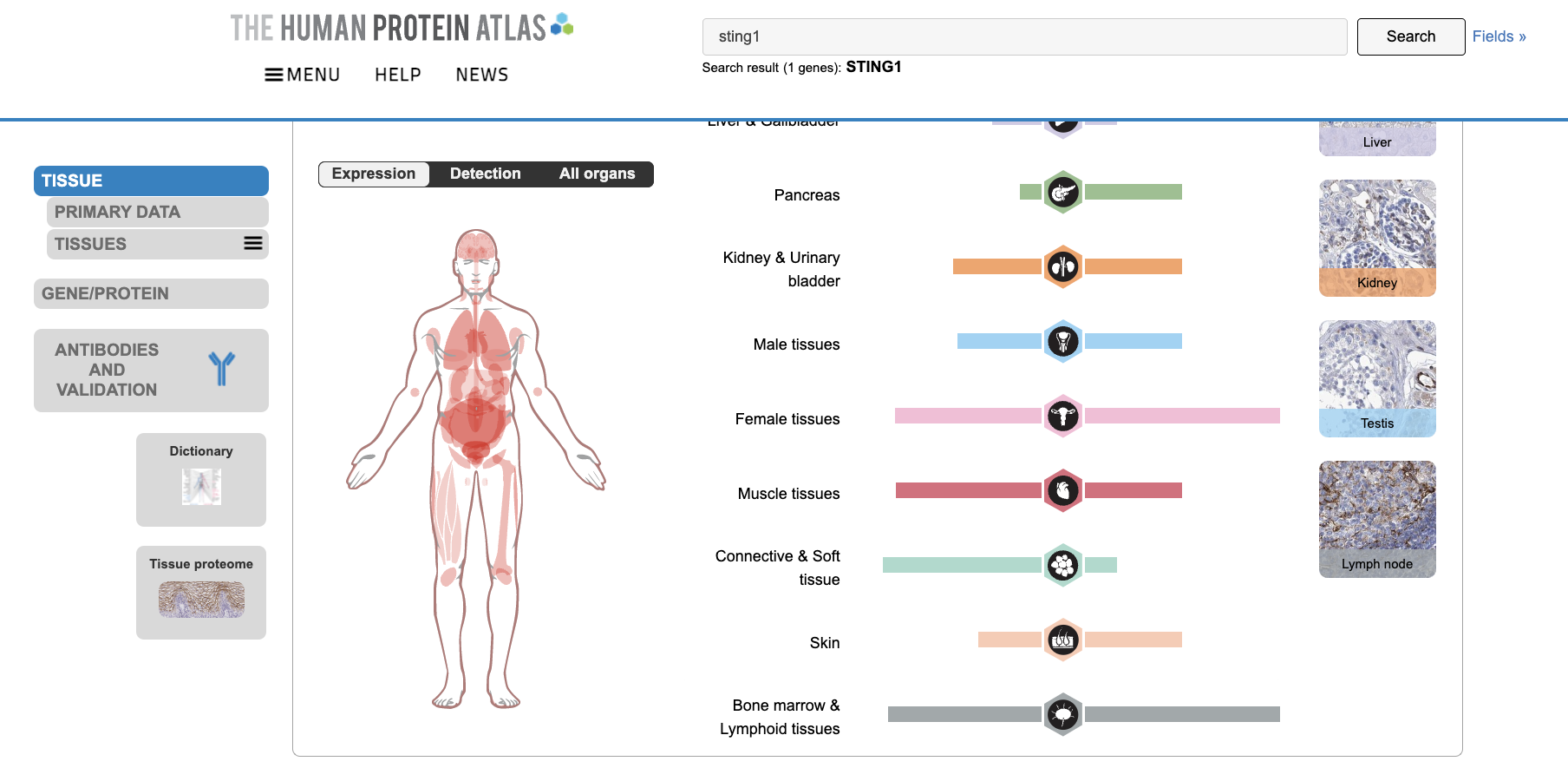


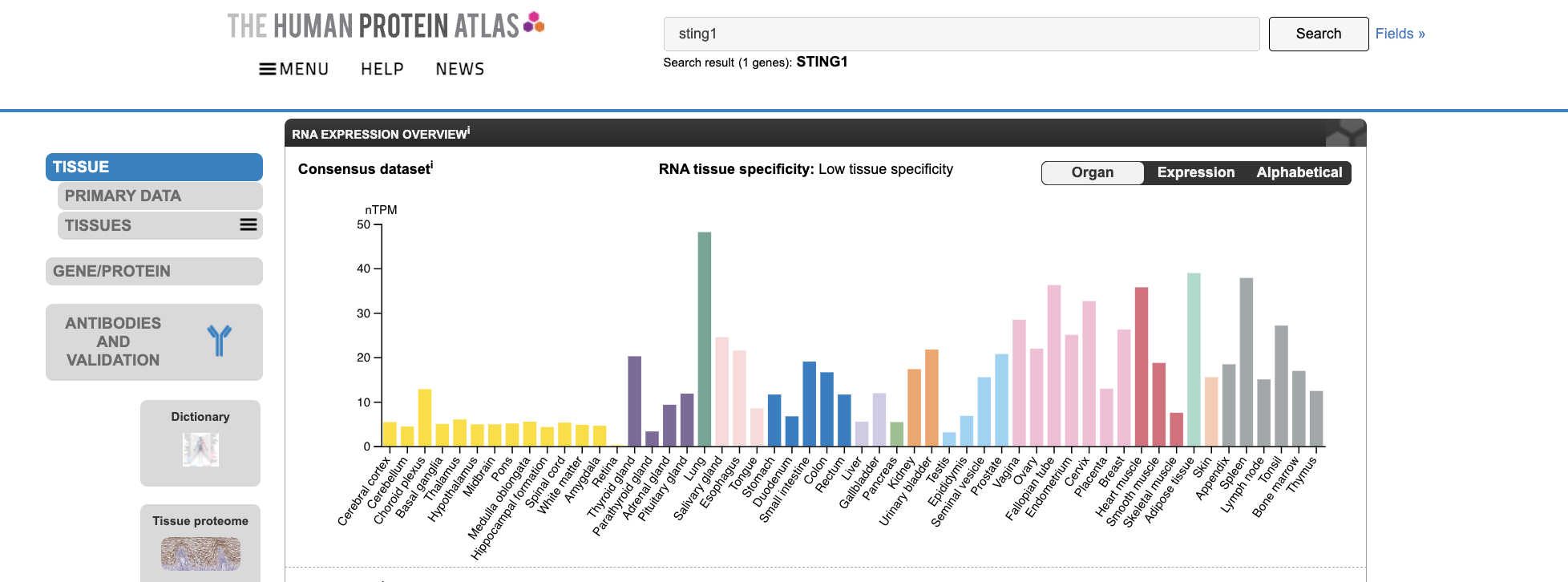
\


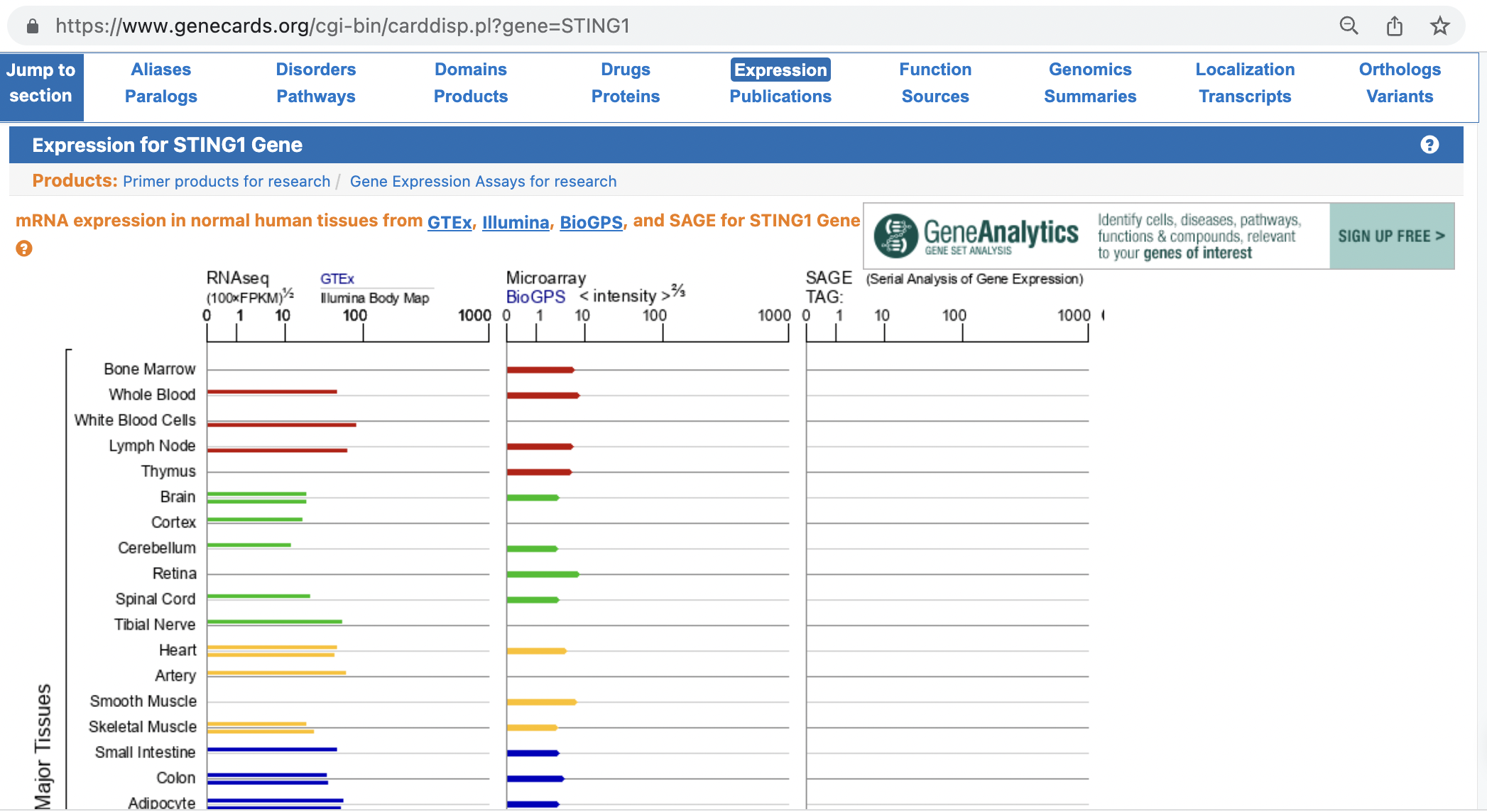


figure S2: **Verification** of the identified candidate gene (TMEM173) expression in skeletal muscle and adipose tissues through; **(A)** Human protein atlas (<https://www.proteinatlas.org/>), and via the **(B)** GeneCards Human Genes database (<https://www.genecards.org/>) so as to decrease the false discovery rate; accessed on May 2022.


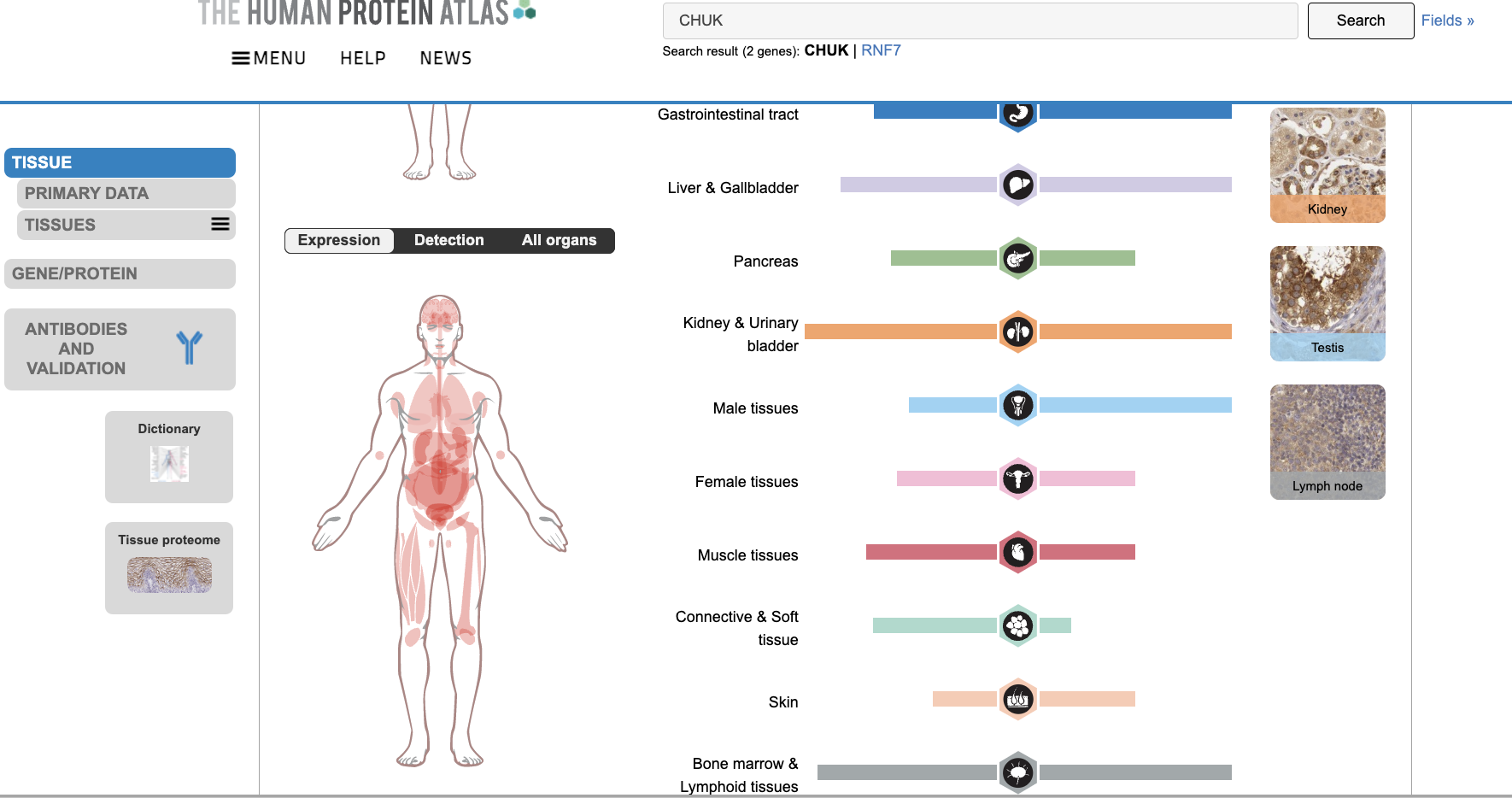


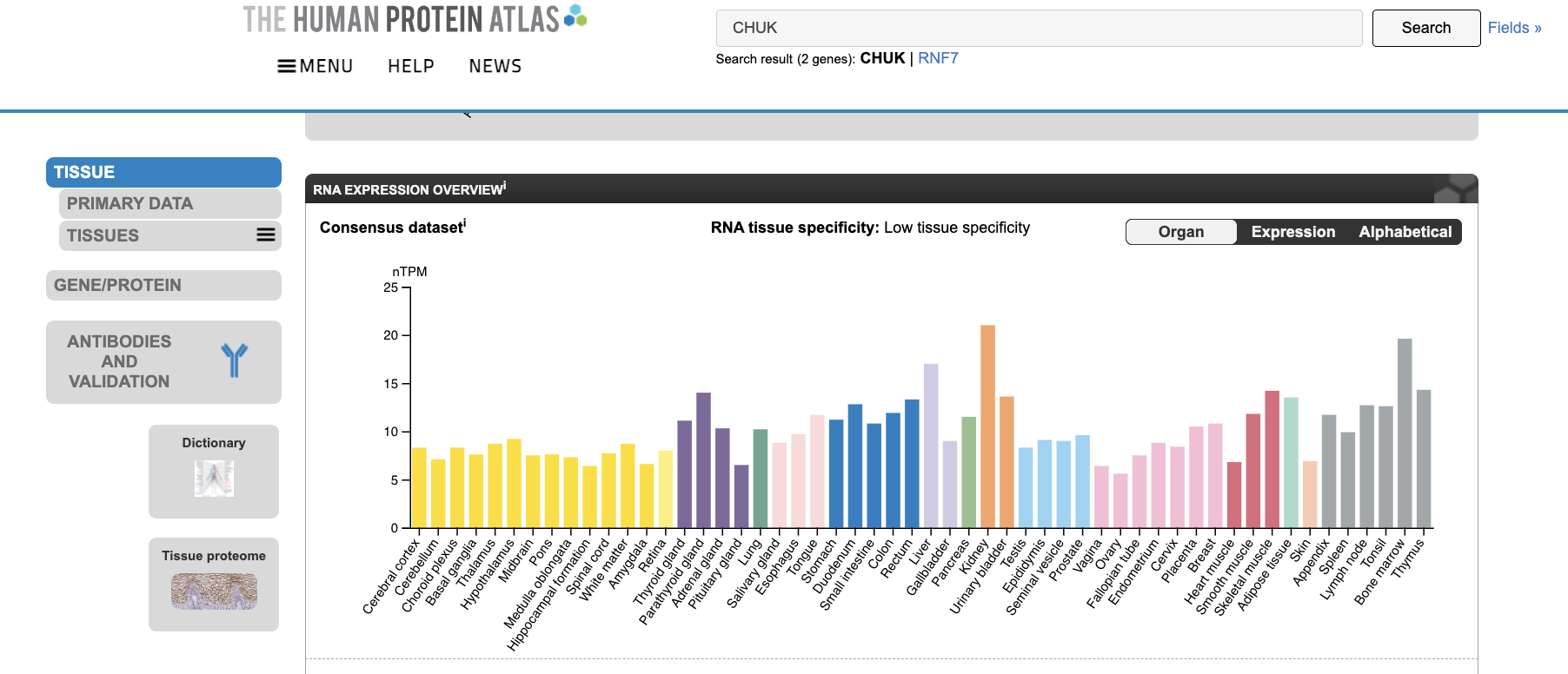


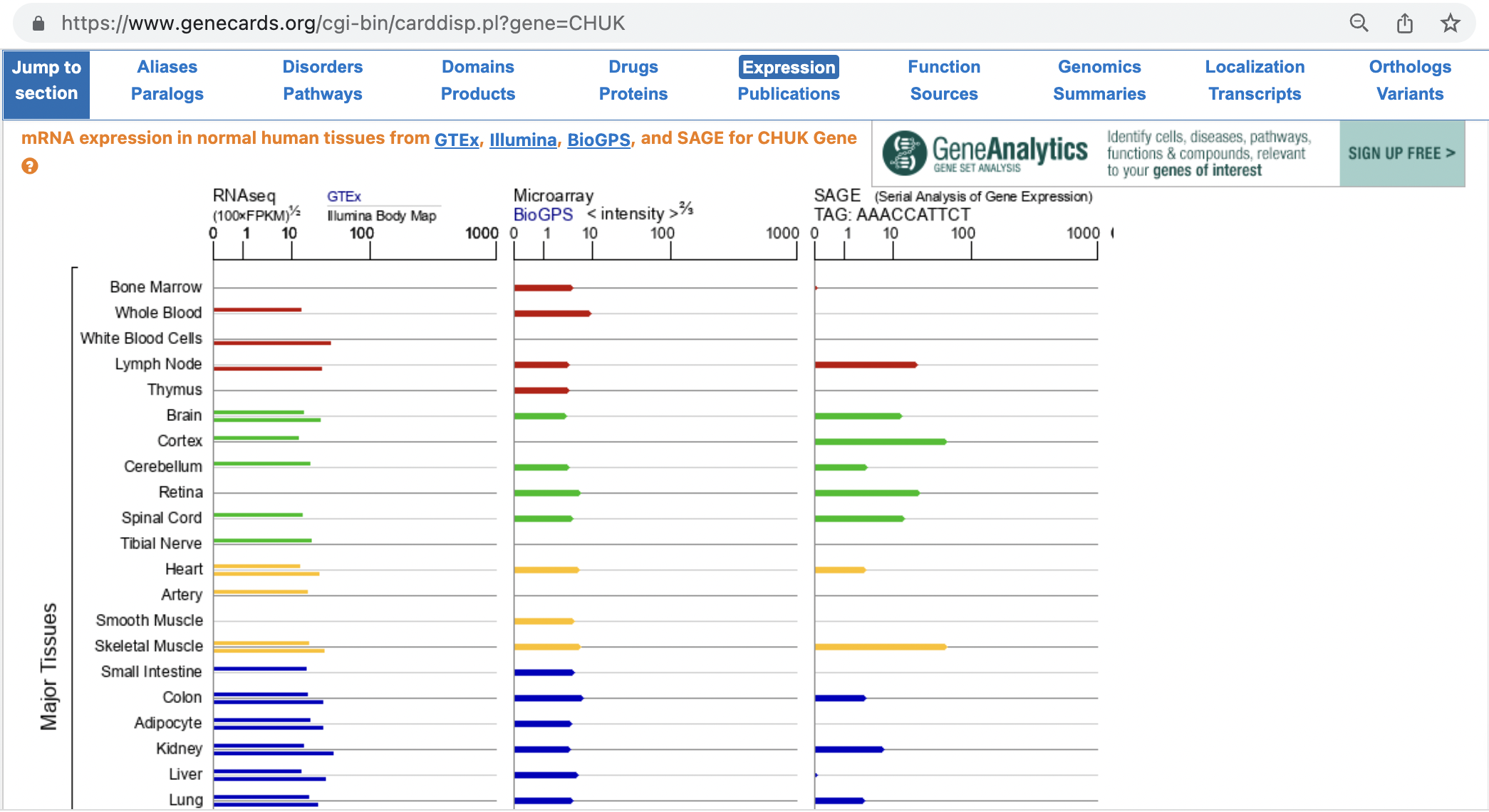


figure S3:**Verification** of the identified candidate gene (CHUK) expression in skeletal muscle and adipose tissues through; **(A)** Human protein atlas (<https://www.proteinatlas.org/>), and via the **(B)** GeneCards Human Genes database (<https://www.genecards.org/>) so as to decrease the false discovery rate; accessed on May 2022.

*
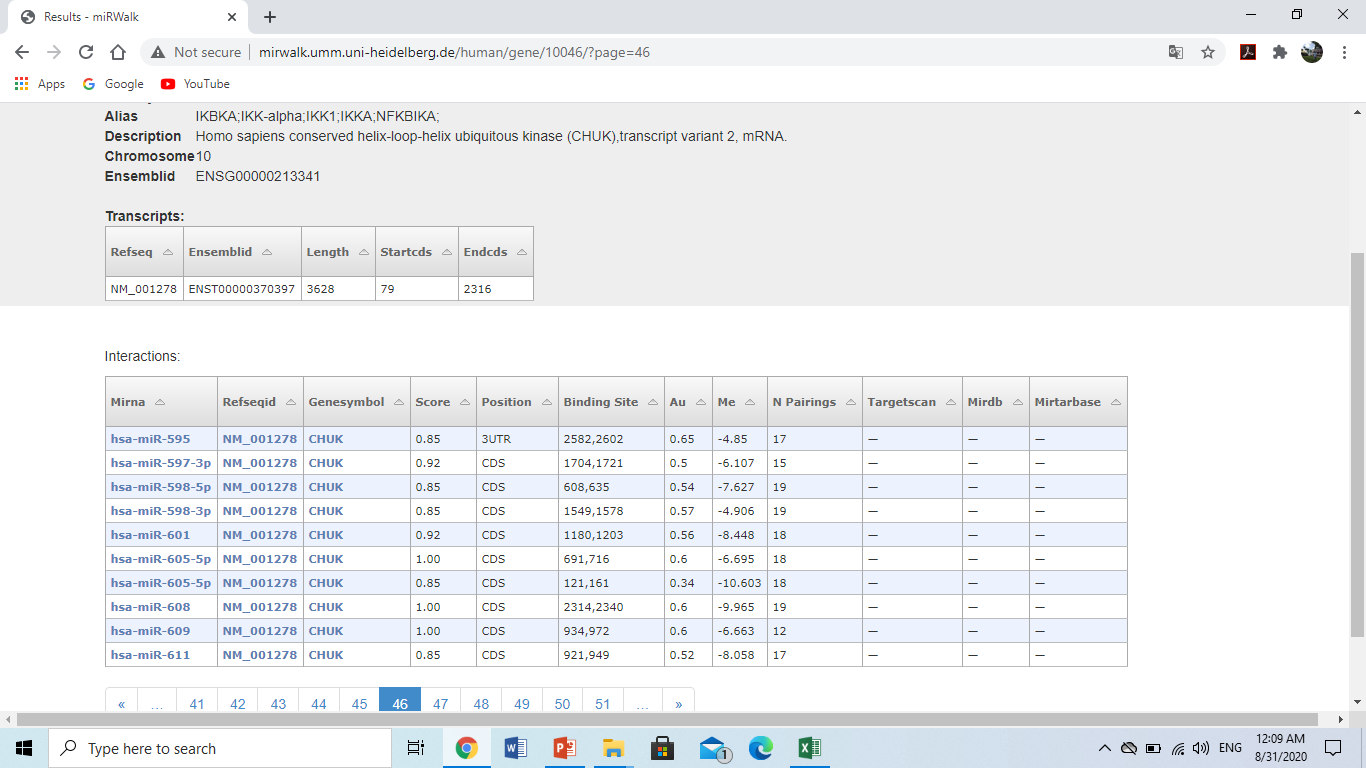
*

**

figure S4: **Retrieval and Verification** of hsa-miR-611 that act as epigenetic regulator for CHUK mRNA in IR; (available at **(A)** <http://mirwalk.umm.uni-heidelberg.de/> & **(B)** <https://www.mirbase.org/>) accessed on May 2022.


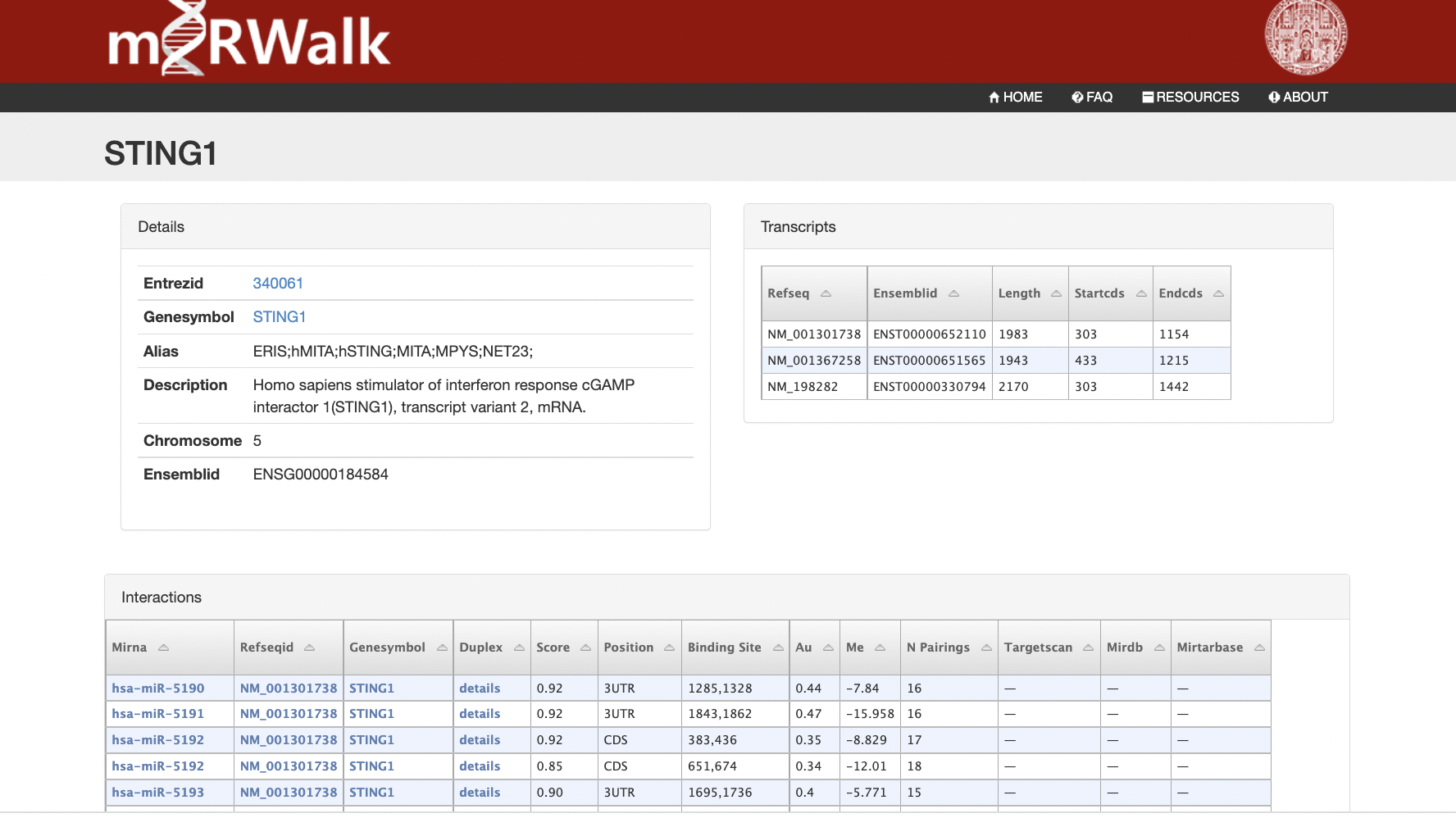


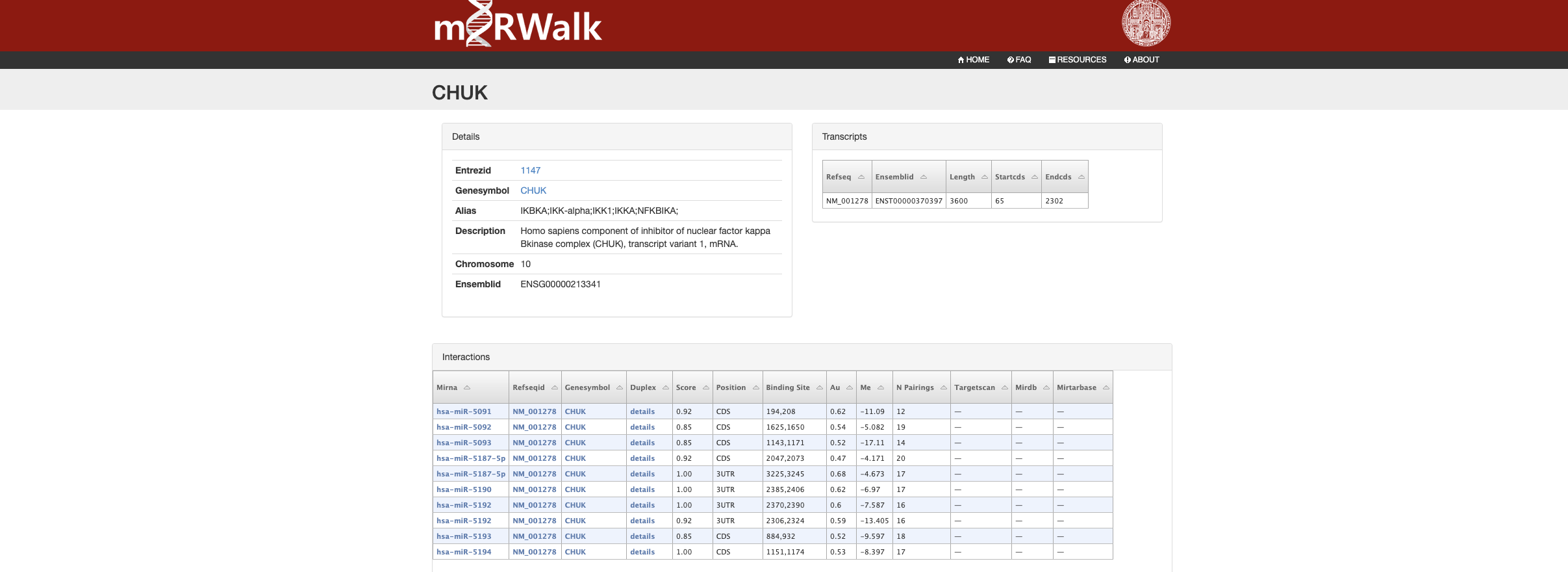


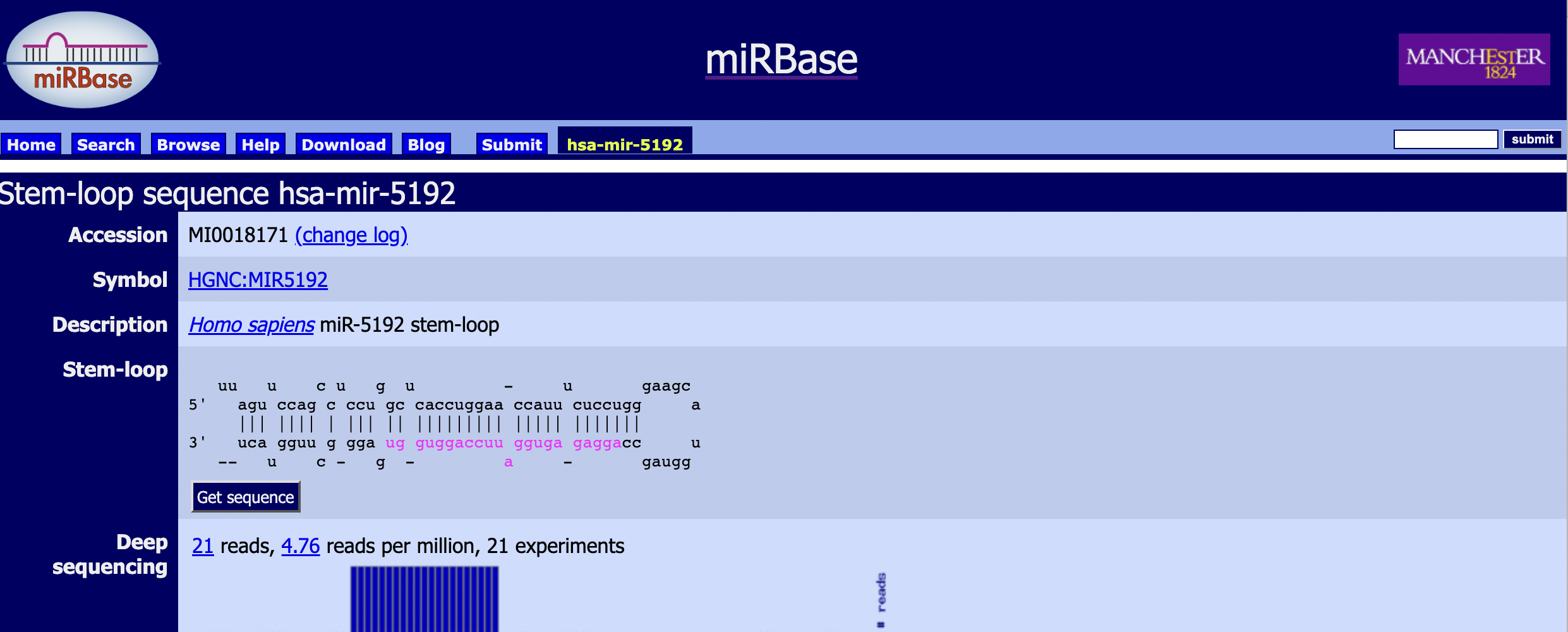


figure S5: **Retrieval and Verification** of hsa-*miR-5192* that act as epigenetic regulator for STING1 & CHUK *mRNAs* in IR; ***(available at (A)*** [*http://mirwalk.umm.uni-heidelberg.de/*](http://mirwalk.umm.uni-heidelberg.de/) & **(B)** <https://www.mirbase.org/>*)* accessed on May 2022.


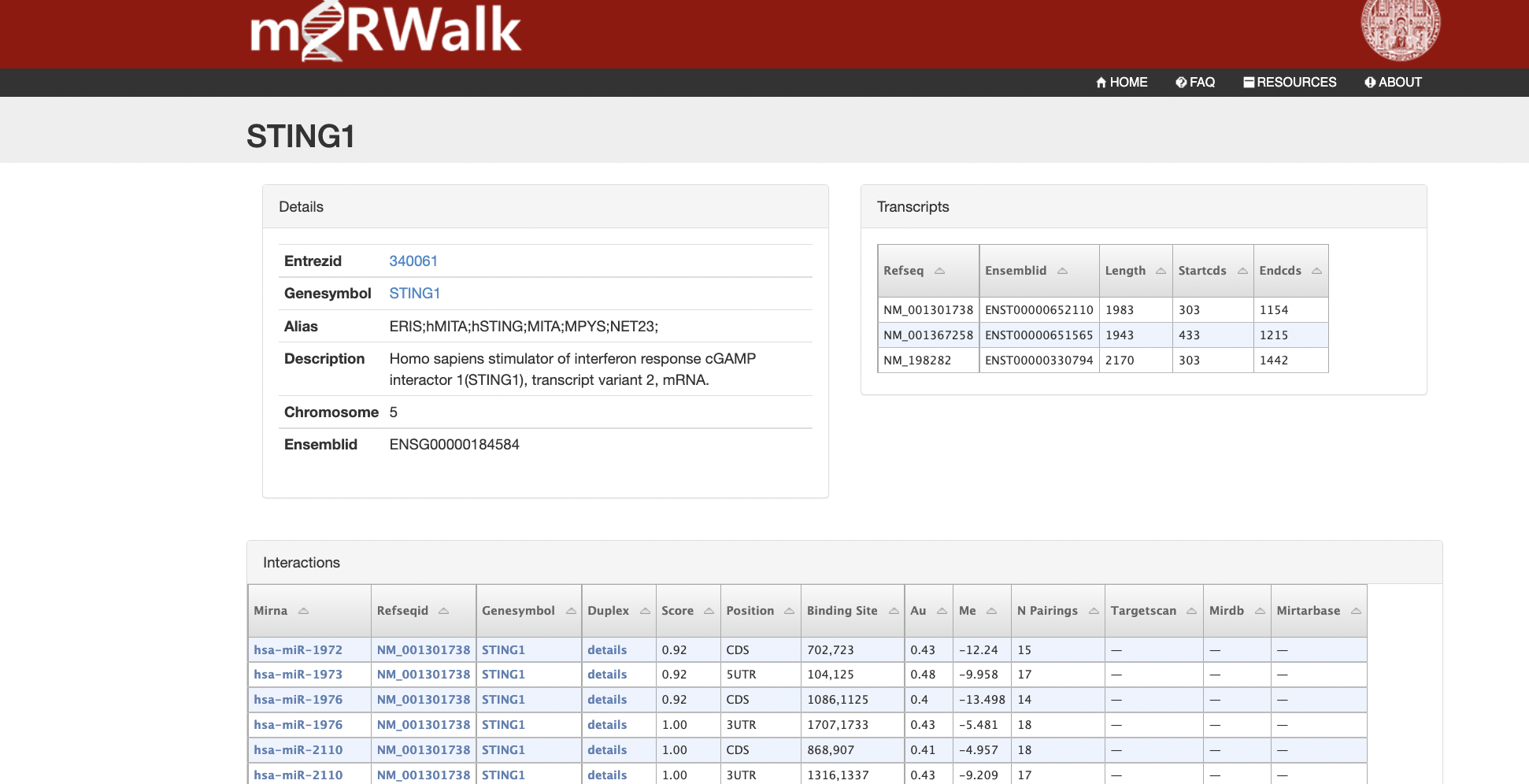


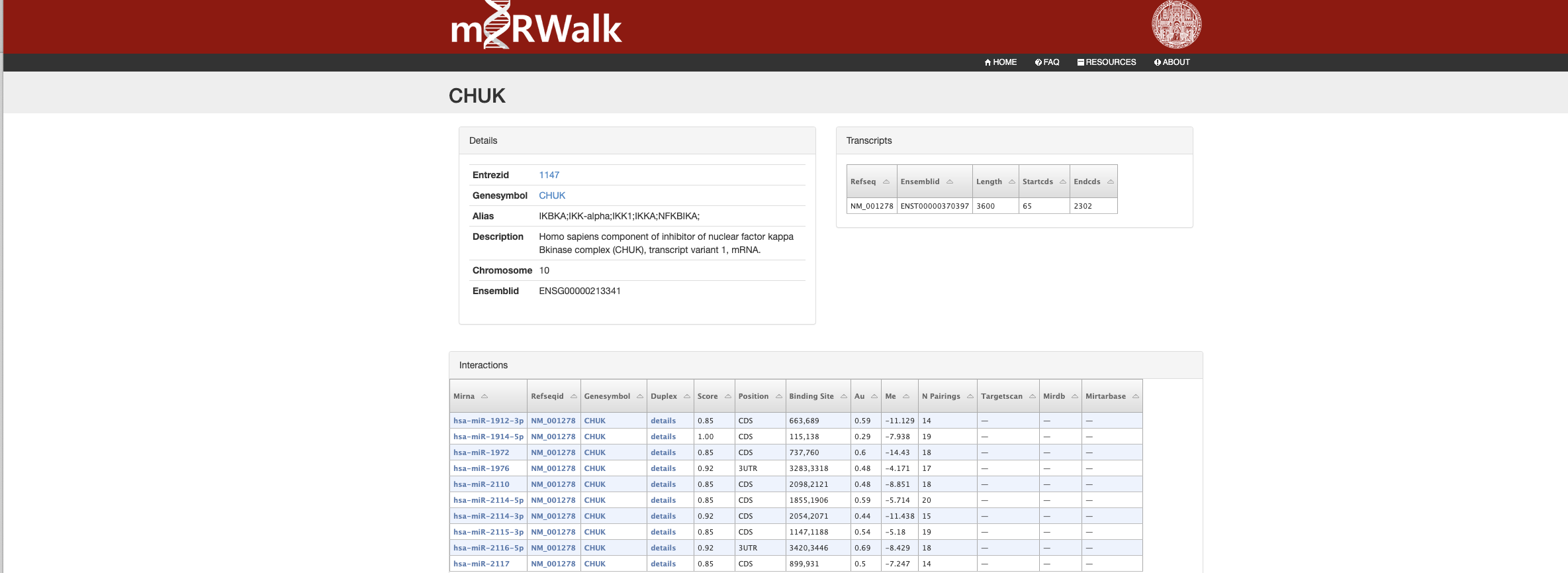


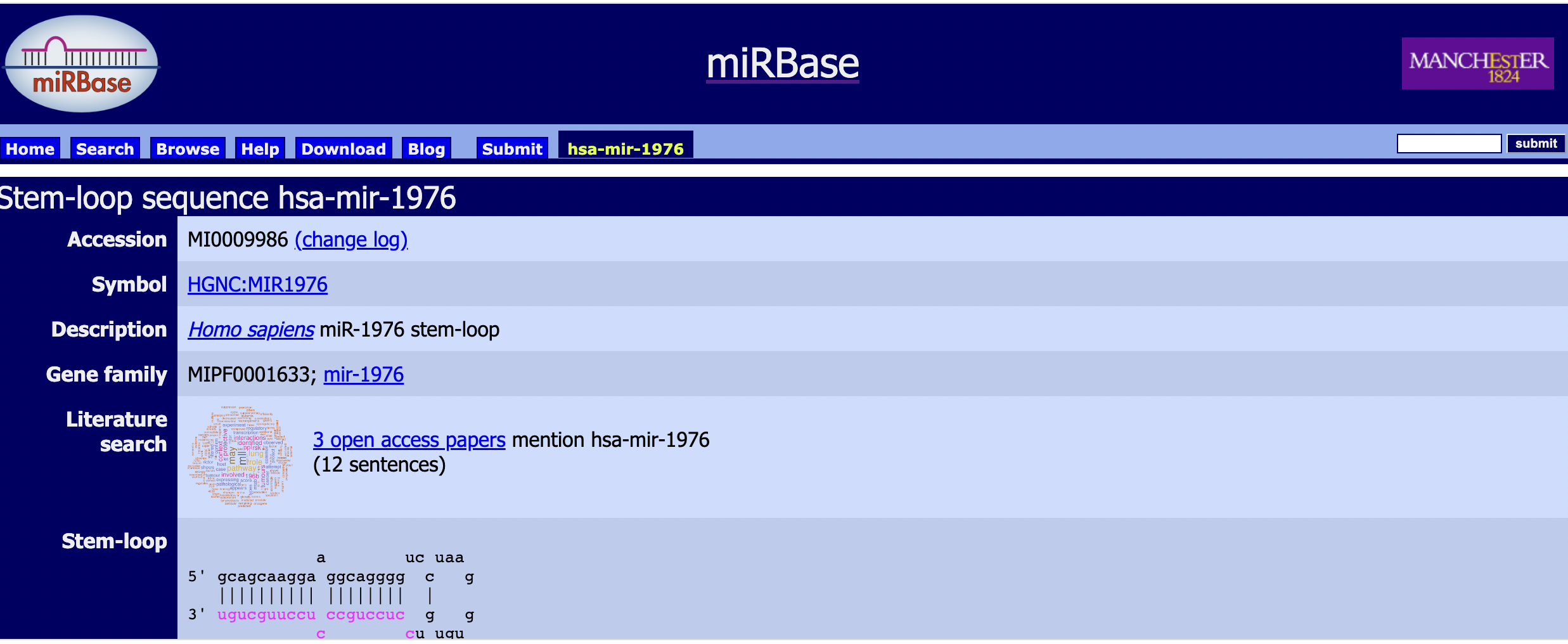


figure S6: **Retrieval and Verification** of hsa-*miR-1976* that act as epigenetic regulator for STING1 & CHUK *mRNAs* in IR; ***(available at (A)*** [*http://mirwalk.umm.uni-heidelberg.de/*](http://mirwalk.umm.uni-heidelberg.de/) & **(B)** <https://www.mirbase.org/>*)* accessed on May 2022.

**

**

**

Figure S7: Retrieval of ***RP4-605O3.4 lncRNA*** from **(A)** **DIANA Tools** database (https://diana.e-ce.uth.gr/lncbasev3) and confirmation of its expression in the interested tissues through **(B) NONCODE** database (http://www.noncode.org/) and **(C)** **LNCipedia** database (https://lncipedia.org/)

*
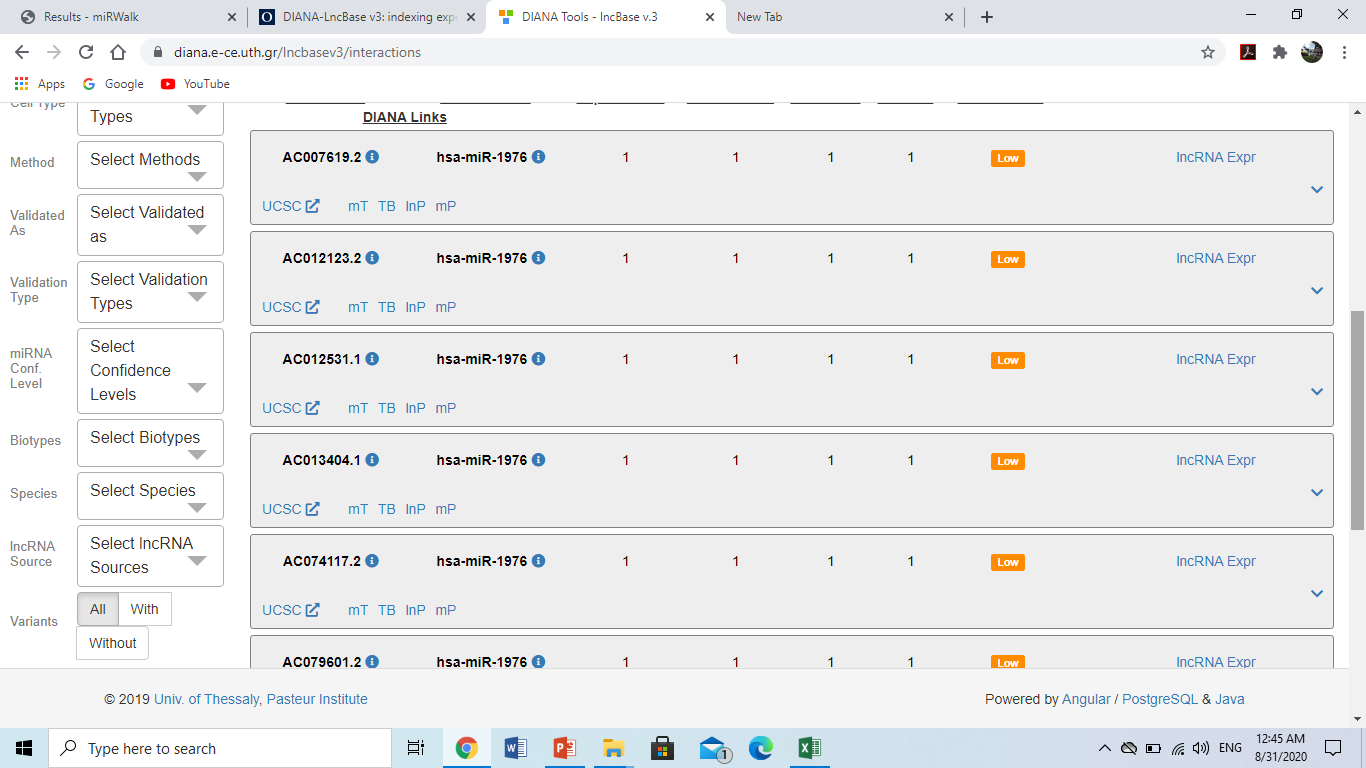
*

**

Figure S8: Retrieval of ***AC074117.2 lncRNA*** from **(A) DIANA Tools** database (https://diana.e-ce.uth.gr/lncbasev3) and confirmation of its expression in the interested tissues through **(B) NONCODE** database (http://www.noncode.org/).

(A) Alignment with *RP4-605O3.4* lncRNA


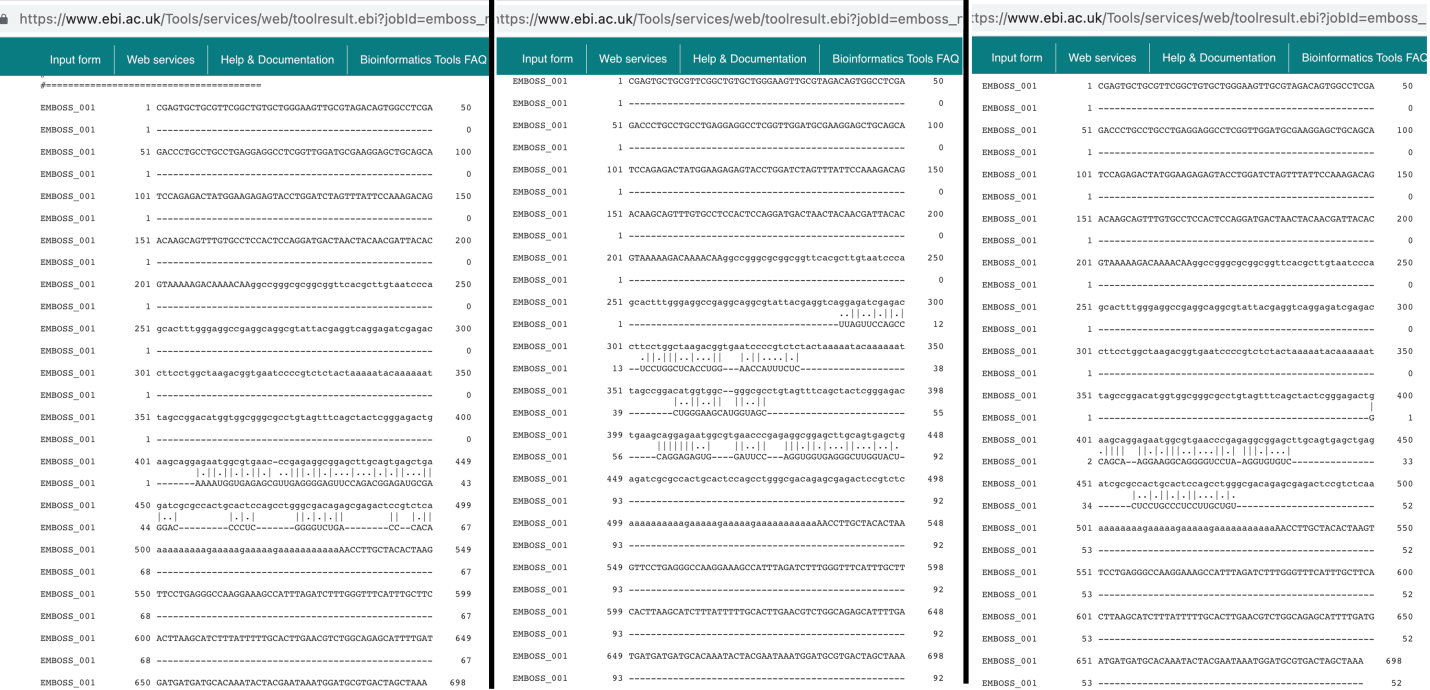


(B) Alignment with *AC074117.2* lncRNA


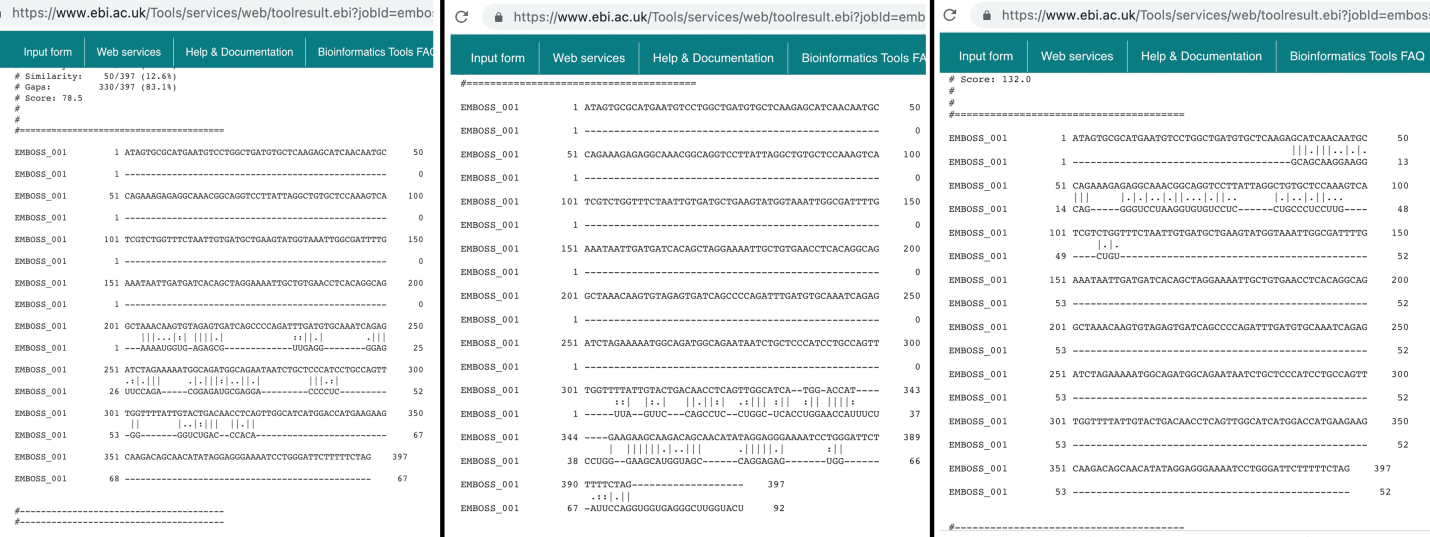


Figure S9: **(A)** Alignment between *RP4-605O3.4* lncRNA and *hsa-miR-611, hsa-miR-5192* & *hsa-miR-1976* miRNAs respectively, **(B)** Alignment between *AC074117.2* lncRNA and *hsa-miR-611, hsa-miR-5192* & *hsa-miR-1976* miRNAs through through EMBL's European Bioinformatics Institute database, Claustal omega database
